# Characterization of Novel Species of Potassium-Dissolving Purple Nonsulfur Bacteria Isolated from In-Dyked Alluvial Upland Soil for Maize Cultivation

**DOI:** 10.3390/life14111461

**Published:** 2024-11-12

**Authors:** Le Thi My Thu, Ly Ngoc Thanh Xuan, Tran Chi Nhan, Le Thanh Quang, Nguyen Duc Trong, Vo Minh Thuan, Tran Trong Khoi Nguyen, Phan Chi Nguyen, Le Vinh Thuc, Nguyen Quoc Khuong

**Affiliations:** 1 Faculty of Crop Science, College of Agriculture, Can Tho University, Can Tho City 900000, Vietnam; thule@ctu.edu.vn (L.T.M.T.); ltquang@ctu.edu.vn (L.T.Q.); nguyenductrongvp4@gmail.com (N.D.T.); thuanvo27022001@gmail.com (V.M.T.); ttknguyen@ctu.edu.vn (T.T.K.N.); lvthuc@ctu.edu.vn (L.V.T.); 2 Experiment and Practice Section, An Giang University, Vietnam National University Ho Chi Minh City, Long Xuyen City 880000, Vietnam; lntxuan@agu.edu.vn (L.N.T.X.); tcnhan@agu.edu.vn (T.C.N.); 3 College of Environment and Natural Resources, Can Tho University, Can Tho City 900000, Vietnam; pcnguyen@ctu.edu.vn

**Keywords:** alluvial soil in dykes, staple crop, plant-growth-promoting substances, potassium solubilization, potassium uptake

## Abstract

Potassium (K) is immobilized within the clay minerals, making it unavailable for plant use. Therefore, the current study aimed to (i) select isolates of purple nonsulfur bacteria that can dissolve K (K-PNSB) and (ii) evaluate the production of plant-growth-promoting substances by the K-PNSB isolates. The results revealed that from in-dyked alluvial soils in hybrid maize fields, 61 K-PNSB isolates were obtained under the pH 5.50 conditions. The total dissolved K content (K_dis_) by the 61 K-PNSB isolates fluctuated from 56.2 to 98.6 mg L^−1^. Therein, three isolates, including M-Sl-09, M-So-11, and M-So-14 had K_dis_ of 48.1–48.8 mg L^−1^ under aerobic dark condition (ADC) and 47.6–49.7 mg L^−1^ under microaerobic light condition (MLC). Moreover, these three isolates can also fix nitrogen (19.1–21.5 mg L^−1^ and 2.64–7.24 mg L^−1^), solubilize Ca-P (44.3–46.8 mg L^−1^ and 0.737–6.965 mg L^−1^), produce indole-3-acetic acid (5.34–7.13 and 2.40–3.23 mg L^−1^), 5-aminolevulinic acid (1.85–2.39 and 1.53–2.47 mg L^−1^), siderophores (1.06–1.52 and 0.92–1.26 mg L^−1^), and exopolymeric substances (18.1–18.8 and 52.0–56.0%), respectively, under ADC and MLC. The bacteria were identified according to their 16S rDNA as *Cereibacter sphaeroides* M-Sl-09, *Rhodopseudomonas thermotolerans* M-So-11, and *Rhodospeudomonas palustris* M-So-14. These potential bacteria should be further investigated as a plant-growth-promoting biofertilizer.

## 1. Introduction

Maize (*Zea mays* L.) is an important cereal used as food for human beings and cattle worldwide [1,2]. In 2022, the maize farming area in the world reached 203.5 million ha with a productivity of 1163.5 million tons [3]. In general, maize yield and growth are heavily affected by chemical fertilizers [4]. For N fertilizer, using 225 kg ha^−1^ of chemical fertilizer, maize yield and dry biomass rose by 23–25% and 14–18%, respectively [5]. Fertilizing P at 8 kg ha^−1^ increased maize grain yield by 15%, protein content by 0.9%, and P uptake by 48% in comparison with that of the negative control [6]. In An Giang province, Vietnam, maize was considered as a main crop, so the strategies to improve fertilizer use efficacy were applied [7]. In agriculture, potassium (K) is an important element in improving the growth and yield of crops. Potassium promotes crop development via metabolisms, such as protein synthesis, photosynthesis, and enzyme activation [8]. K exists in soil in dissolved, exchangeable, non-exchangeable, and mineral forms [9]. However, only 1–2% of soil K can be absorbed by crops, while 90–98% of soil K is immobilized in silicate minerals [10]. The concentration of exchangeable K is extremely low because most of the K content in the soil is in an insoluble form [11]. In addition, the presence of clay minerals affects the release of non-exchangeable and K availability [12]. Moreover, fast-growing agriculture can reduce the soil K due to plant uptake, erosion, and leaching [13]. Thus, K-dissolving microorganisms play an important role in dissolving K to provide exchangeable K for crops [14].

K-dissolving microorganisms have been used to dissolve K from different K minerals to increase soil K content [15]. Among them, the application of K-dissolving bacteria (KDB) has been known as an approach to increase K availability in K-deficient soils [16]. However, the application use of the beneficial bacteria is limited due to their poor penetrance to plant roots and adaptability to different environments [17]. In the meantime, purple nonsulfur bacteria (PNSB) have been applied for crop production because they can produce and accumulate beneficial metabolites for plants [18]. Purple nonsulfur bacteria can fix nitrogen [19], solubilize phosphorus [20], dissolve K [21], remove heavy metals [22], and reduce CH_4_ emission [19,23]. Moreover, PNSB can secrete plant-growth-promoting substances such as indole-3-acetic acid (IAA), and 5-aminolevulinic acid (ALA) to facilitate plant growth [23,24]. Simultaneously, this is also an approach to reduce chemical fertilizer use. However, there have not been any studies investigating dissolved K content (K_dis_) by PNSB isolates in upland soils. Therefore, this study was performed to select potential K-dissolving PNSB (K-PNSB) for applications on upland crops.

## 2. Materials and Methods

### 2.1. Materials

The basic isolation medium (BIM) was prepared according to Brown [25] to isolate PNSB in a Petri dish (90 mm × 15 mm; Dinlab, Casablanca, Morocco). The preparation in 1 L of distilled water included 1.0 g (NH_4_)_2_SO_4_ (Xilong, Xinjiang, China) 0.5 g K_2_HPO_4_ (Xilong, Xinjiang, China), 0.2 g MgSO_4_.H_2_O (Xilong, Xinjiang, China), 2.0 g NaCl (Xilong, Xinjiang, China), 5.0 g NaHCO_3_ (Xilong, Xinjiang, China), 1.5 g yeast extract (Angel, Yichang, China), 1.5 g glycerol (Xilong, Xinjiang, China), and 0.03 g L-cysteine (Shanghai Lanji Technology Development, Shanghai, China). The solid BIM was added with 15 g agar (Hai Long, Ho Chi Minh City, Vietnam) to purify bacterial isolates.

### 2.2. Methods

#### 2.2.1. Isolation of PNSB from Hybrid Maize Fields

Sampling methods were conducted in each maize field of the vegetative stage at 45 days after sowing in communes of Phu Hoi, Nhon Hoi, and Quoc Thai of An Phu District, An Giang province. Soil, slurry, and water were collected from 5 different positions at a depth of 0–10 cm from the soil surface, 0–5 cm from the slurry surface, and 5–10 cm from the water surface, respectively (Figure S1). The hybrid maize variety was DK6919S (Bayer Company, Leverkusen, Germany). Different sites of a sample were combined as a representative sample for the maize field, including 0.5 kg soil, 0.5 kg mud, and 250 mL water. Sampling tools (Eijkelkamp, Giesbeek, The Netherlands) were sanitized by alcohol 96% before use. The samples were kept in clean plastic bags, tied, and vacuumed. The samples were stored at 4 °C until isolation.

Isolation methods: 1.0 g of soil or slurry was tested in a test tube (Biohall, Haryana, India) containing 18 mL BIM pH = 7.0 sealed by 1.5 mL sterilized paraffin (Xilong, Xinjiang, China) to ensure anaerobic condition. On the other hand, 9 mL of water was tested in a test tube containing 9.0 mL BIM (pH = 7.0), also sealed by 1.5 mL paraffin.

Then, all of the samples were cultured under continuous illumination at 3000 lux at room temperature (25–40 °C) (microaerobic light condition, MLC) for 5–7 days. When the culture turned red, pink, brown, or brownish yellow, the culture was spread on solid BIM pH = 7.0 until monocolonies appeared. Then, all of the dishes were incubated in clear anaerobic jars (Thermo Scientific, Waltham, MA, USA) facilitated by AnaeroPack-CO_2_ MGC (Visitech, Tokyo, Japan) to remove oxygen. The pure colonies were held under MLC [26].

Soil analytic methods: The collected soil was analyzed according to Sparks et al. [27].

#### 2.2.2. Selection of PNSB

Bacteria preparation: The pure bacteria were cultured under MLC for 48 h and adjusted to the density of 1 × 10^8^ CFU mL^−1^ corresponding to OD = 0.5 at 660 nm wavelength under pH = 5.5 condition.

Medium preparation: Liquid BIM after autoclave was adjusted to pH = 5.5. Based on the experiment, a suitable precursor was added. Therein, K_n_Na_12−n_[(AlO_2_)_12_(SiO_2_)_12_]•xH_2_O (SIGALD, Darmstadt, Germany), Ca_3_(PO_4_)_2_ (JHD, Shaanxi, China) tryptophan (Sisco Research Laboratories, Andheri, India), glycine (Zhanyun, Shanghai, China), sodium acetate (Xilong, Xinjiang, China), succinate (Merck, Darmstadt, Germany), and FeCl_3_•6H_2_O (Xilong, Xinjiang, China) were autoclaved separately from the medium and added to BIM before use. All of the BIM, distilled water, reagents, and equipment were autoclaved for 20 min at 121 °C and 1 atm before use. After that, the pH was adjusted to pH = 5.50 by HCl 1.0 M (Xilong, Xinjiang, China) and filtered by microbial filter membrane (0.45 µm) in the safety cabinet. The pH = 5.50 was found in the in-dyked alluvial soil.

Evaluation: The ratio between the liquid BIM and suspension of the selected K-PNSB isolates was 90 and 10% in volume, respectively. Under the aerobic dark condition (ADC), a 50 mL centrifugal tube (BIOLOGIX, Camarillo, CA, USA) was shaken by a reciprocal shaker (Labtron, Camberley, UK) at 150 rpm at 30 °C, while under MLC, a 12 mL test tube was used.

This experiment was completely randomized on liquid BIM added with 0.5 g L^−1^ K_n_Na_12−n_[(AlO_2_)_12_(SiO_2_)_12_]•xH_2_O under both MLC and ADC. After 72 h of incubation, the culture was centrifuged at 3500 rpm for 15 min. After that, the solution was measured by atomic absorption spectroscopy (Shimadzu, Kyoto, Japan) at 766.5 nm wavelength to measure for the K_dis,_ according to Sparks et al. [27].

#### 2.2.3. Evaluation of Nitrogen Fixation, Phosphate Solubilization, and Synthesis of IAA, Siderophores, and ALA by the K-PNSB

Experiment 1: Nitrogen fixation by the K-PNSB under pH 5.5: This experiment was completely randomized on liquid BIM without N. After 72 h of incubation under MLC and ADC, the culture was centrifuged at 3500 rpm for 15 min. Then, the solution was measured by spectrophotometry (Shimadzu, Kyoto, Japan) at 650 nm wavelength for the fixed N content according to the color of blue phenol [28].

Experiment 2: Phosphate solubilization by the K-PNSB: This experiment was completely randomized on liquid BIM, but K_2_HPO_4_ was replaced by 0.5 g L^−1^ of an insoluble P compound Ca_3_(PO_4_)_2_. After 120 h of incubation under MLC and ADC, the culture was centrifuged at 3500 rpm for 15 min. Then, the solution was measured by spectrophotometry at 880 nm wavelength for the solubilized P content according to the ascorbic acid (Xilong, Xinjiang, China) method [29].

Experiment 3: Indole-3-acetic acid production by K-PNSB from tryptophan: This experiment was completely randomized on liquid BIM added with 100 mg L^−1^ tryptophan. After 72 h of incubation under MLC and ADC, the culture was centrifuged at 3500 rpm for 15 min. Then, the IAA was measured by Salkowski colorimetry. The steps were as follows: 0.74 mL of centrifuged bacterial culture was mixed with 3 mL of Salkowski reagent (4.5 g L^−1^ FeCl_3_ in 10.8 M H_2_SO_4_ (Xilong, Xinjiang, China). After 20 min of incubation, the IAA was measured by spectrophotometry at 535 nm wavelength [30].

Experiment 4: 5-aminolevulinic acid production by K-PNSB: This experiment was completely randomized on liquid BIM containing ALA precursors, including glycine (0.563 g L^−1^) and sodium acetate (5.44 g L^−1^). After 96 h of incubation under MLC and ADC, the culture was centrifuged at 3500 rpm for 15 min. Then, ALA was measured according to Burnham [31]. In particular, 0.5 mL of the centrifuged bacterial culture was mixed with 0.15 mL of an assimilating mixture (2 mL ATP 0.2 M (Merck, Darmstadt, Germany), 1.75 mL CoA 0.01 M (Merck, Darmstadt, Germany), 1.35 mL pyridoxal phosphate 0.01 M (Biosynth Ltd., Compton, UK), and 2 mL distilled water) and 0.35 mL of substrates (5 mL glycine 1.0 M, 5 mL succinate 1.0 M, 5 mL MgCl_2_ 0.1 M (Xilong, Xinjiang, China), and 2.5 mL Tris-HCl buffer 1M (Sisco Research Laboratories, Andheri, India) and incubated at 37 °C for 30 min. Next, 0.5 mL of trichloroacetic acid (Xilong, Xinjiang, China) was added to stabilize for 5 min, then transferred to a test tube containing 2 mL acetate buffer 1.0 M (Xilong, Xinjiang, China) pH 4.7 and 2 drops of acetylacetone (Xilong, Xinjiang, China), and incubated for 15 min at 99 °C. Then, the solution was measured at 553 nm wavelength. The Ehrlich consisted of 1 g P-dimethyl-amino benzaldehyde (Merck, Darmstadt, Germany) in 30 mL glacial acetic acid (Xilong, Xinjiang, China) and 8 mL 70% perchloric acid (Alpha Chemika, Andheri, India) and adjusted to 50 mL by glacial acetic acid.

Experiment 5: Siderophores production by K-PNSB: This experiment was completely randomized on liquid BIM added with siderophores precursors, including 1 g L^−1^ succinate and 0.5 µM FeCl_3_•6H_2_O. After 96 h of incubation under MLC and ADC, the culture was centrifuged at 3500 rpm for 15 min. Then, 0.5 mL of the centrifuged bacterial culture was mixed with 0.5 mL of an indicator and measured at 553 nm by spectrophotometry [32]. The indicator consisted of 6 mL hexadecyl trimethyl amoni 10 mM (TNJ, Anhui, China), 1.5 mL FeCl_3_•6H_2_O 1 mM (in HCl 10 mM), 7.5 mL chrome azurol S 2 mM (Merck, Darmstadt, Germany). Next, 4.307 g of Anhydrous piperazine (Merck, Darmstadt, Germany) was added and adjusted to pH = 5.6. Then, distilled water was added, shaken, added with 0.1017 g 5-sulfosalicylic acid (Xilong, Xinjiang, China), shaken again by hand for complete dissolution, and added with distilled water to the volume of 100 mL.

Experiment 6: Exopolymeric substances (EPS) production by K-PNSB: This experiment was completely randomized on liquid BIM. The bacteria were cultured in BIM for 72 h under MLC and ADC. Then, the culture was centrifuged at 3500 rpm for 15 min to separate the cell biomass and supernatants. Therein, the supernatants were used to extract EPS. The EPS was collected according to the modified method of Eboigbodin and Biggs [33] as follows: the supernatants were mixed with cold ethanol (Xilong, Xinjiang, China) (4 °C) at the ratio of 1:2.2 and incubated for 24 h at −20 °C to precipitate EPS. After that, the mixture was centrifuged at 3500 rpm for 15 min at 4 °C to collect EPS, which was then dried at 45 °C and weighed [34].

#### 2.2.4. Identification of K-PNSB

The bacterial isolates were cultured in BIM pH 5.5 under MLC for 48 h to be later identified by 16S rRNA technique. Additionally, 2 mL of each bacterial culture was centrifuged at 10,000 rpm for 5 min to collect cells to extract DNA. The cell pellet was washed with ethanol 70% and 90% for 5 min, respectively. After each wash, the mixture was centrifuged at 10,000 rpm for 5 min. The DNA was dissolved in 1X TE buffer (Merck, Darmstadt, Germany). The purity of the DNA was checked by electrophoresis in 1.0% *w*/*v* agarose gel (Merck, Darmstadt, Germany) under UV light. The 16S rDNA sequence of the bacteria was amplified by polymerase chain reaction with the following primer pair: 16S Forward Primer 8 F (5′-AGA GTT TGA TCC TGG CTC AG-3′) and 16S Reverse Primer 1492 R (3′-GGT TAC CTT GTT ACG ACT T-5′). The thermal cycle was as follows: The first DNA denaturation started at 95 °C for 5 min, a 30-time-cycle consisted of 95 °C for 30 s, 55 °C for 30 s, and 72 °C for 2 min, the last elongation was at 72 °C for 10 min, and the termination was at room temperature (BioRad, Hercules, CA, USA). The amplicons were purified by the TIANquick Midi tool kit, according to the manufacturer. The amplicons were checked by electrophoresis in 1.0% *w*/*v* agarose gel under UV light. They were then sequenced by the Macrogen DNA Sequencing Service (Macrogen, Seoul, Republic of Korea). The results were analyzed by BioEdit version 7.0.5.3 [35] and ChromasPro version 1.7 (http://technelysium.com.au/wp/chromaspro; accessed on 10 May 2024) and compared with the available sequences in the GenBank database by BLAST in NCBI.

#### 2.2.5. Statistical Analysis

The data and graphs were processed by Microsoft Office Excel 2016. The data were tested for normal distribution before they were used to run significance at a *p*-value of 0.05. The statistical comparison was performed using ANOVA and a Duncan test in SPSS version 20.0.

## 3. Results

### 3.1. Potassium-Dissolving Purple Nonsulfur Bacteria Isolation

#### 3.1.1. Traits of Soils That Were Used to Isolate K-PNSB

Table 1 reveals that the average actual acidity (pH_H2O_) of the 30 locations of K-PNSB collection was 5.73, while the potential acidity (pH_KCl_) was 5.05 ± 0.15. EC fluctuated from 0.15 to 0.88 µS cm^−1^. Furthermore, the soil CEC was 19.3 ± 0.36 meq 100 g^−1^. The average exchangeable K in the soil was 0.27 ± 0.02 meq 100 g^−1^. The total N and total P in the soil were 0.114 ± 0.005 and 0.089 ± 0.004%, respectively. Moreover, the available N and soluble P ranged from 2.86 to 28.7 and 27.8 to 243.6 mg kg^−1^, respectively. Moreover, the insoluble Ca-P in the soil was 188.0 ± 8.57 mg kg^−1^.

#### 3.1.2. Isolation of K-PNSB

From 30 soil samples, 30 slurry samples, and 30 water samples collected from the hybrid maize fields, 61 PNSB isolates were obtained under pH 5.50. The K-PNSB isolates were brown, brownish-yellow, red, and purple (Figure S2). Under MLC, the K_dis_ fluctuated from 26.9 to 48.8 mg L^−1^, while under ADC, the number was 26.1–49.7 mg L^−1^, resulting in the total K_dis_ by the 61 K-PNSB isolates from 56.2 to 98.6 mg L^−1^ (Table S1). Moreover, the M-So-11 showed the greatest K dissolution under each MLC and ADC. However, K-PNSB isolates that had low K-dissolving capacity were less efficient under field conditions. Thus, K-PNSB isolates with a total K_dis_ lower than 70 mg L^−1^ under both conditions were eliminated from the next experiments. In other words, 48 out of 61 isolates were selected.

### 3.2. Selection of K-PNSB That Can Synthesize Plant-Growth-Promoting Substances

#### 3.2.1. Selection of K-PNSB

According to Table 2, under ADC, isolates M-So-11 and M-So-14 can dissolve K, with K_dis_ content of 48.8 mg L^−1,^ which was equivalent to those of the isolates M-Sl-02, M-Sl-09, M-Sl-12, M-So-10, M-So-25, and M-Wa-07 (roughly 46.8–48.5 mg L^−1^). On the other hand, under MLC, the isolates M-Wa-03, M-Wa-05, M-Wa-15, M-Wa-19, and M-Wa-25 had a low K-dissolving capacity of roughly 35.0–37.6 mg L^−1^. The other PNSB isolates can dissolve K from 37.7 to 45.9 mg L^−1^. On the other hand, under MLC, isolates M-Sl-09 and M-So-11 had K_dis_ of 49.6 and 49.7 mg L^−1^, which were statistically equivalent to those of isolates M-Sl-01, M-So-07, M-So-14, and M-Wa-11 (K_dis_ = 47.5–49.0 mg L^−1^). The isolates M-Wa-05, M-Wa-06, and M-Wa-10 poorly dissolved K with K_dis_ of 32.5, 31.5, and 30.7 mg L^−1^, respectively. Moreover, the other isolates dissolved at 33.5–46.5 mg L^−1^ (Table 2). Among them, there were 28 isolates and 23 isolates that had K dissolution over 40 mg L^−1^ under ADC and MLC, respectively. Thus, isolates that had K_dis_ over 40 mg L^−1^ under both incubating conditions were selected for further experiments. In other words, the 22 K-PNSB isolates can greatly dissolve K under both ADC and MLC, and they were chosen for the test for nitrogen fixation and Ca-P solubilization (Table 2).

#### 3.2.2. Ability of Nitrogen Fixation and Phosphate Solubilization of K-PNSB Isolates

**Figure 1:**
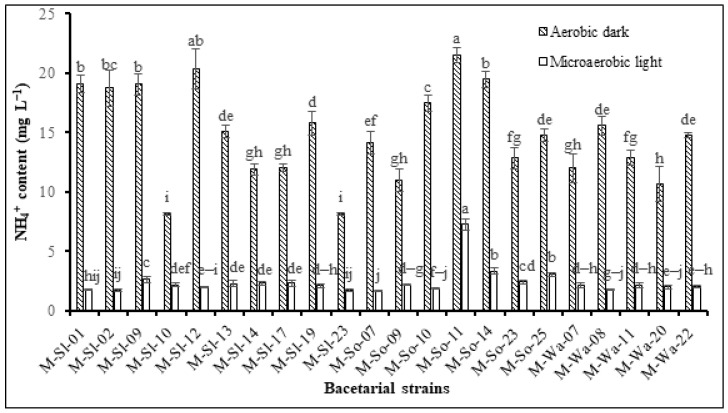
Nitrogen fixation of potassium-dissolving purple nonsulfur bacteria selected from in-dyked alluvial soil in hybrid maize fields. Note: Different letters indicate a significant difference (*p* < 0.05).

Overall, the ADC exhibited a better N-fixing condition than the MLC. The isolate M-So-11 greatly fixed nitrogen under ADC, with equivalent NH_4_^+^ produced at 21.5 mg L^−1^ to the isolates M-Sl-12. In addition, isolates M-So-14, M-Sl-09, M-Sl-01, and M-Sl-02 also had equivalent NH_4_^+^ concentrations with 19.5 and 19.1, 19.1, and 18.8 mg L^−1^. The isolates M-Sl-10 and M-Sl-23 had the lowest nitrogen fixation with NH_4_^+^ 8.16 mg L^−1^, while the other isolates resulted from 10.7 to 17.5 mg NH_4_^+^ L^−1^ (Figure 1). Under MLC, the isolates M-So-11 had the greatest nitrogen fixation with 7.24 mg NH_4_^+^ L^−1^. Subsequently, the isolates M-So-14, M-So-25, and M-Sl-09 had NH_4_^+^ concentrations of 3.30, 3.05, and 2.64 mg L^−1^, respectively. The isolates M-Sl-23, M-Sl-02, and M-So-07 poorly fixed nitrogen, with NH_4_^+^ of 1.67, 1.67, and 1.66 mg L^−1^, respectively (Figure 1).

**Figure 2:**
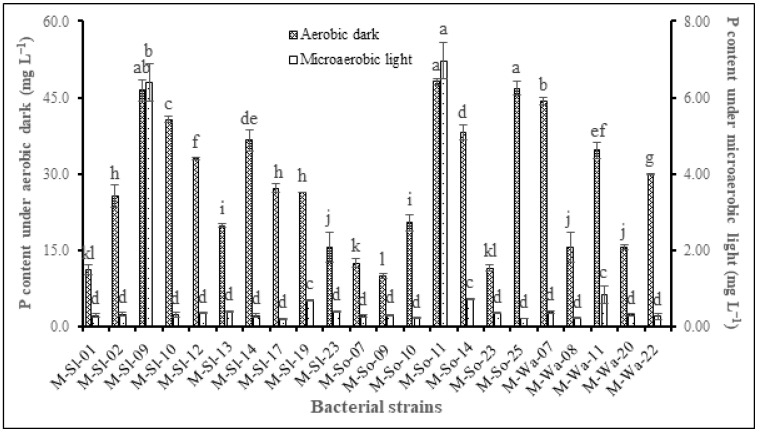
Ca-P dissolution of potassium-dissolving purple nonsulfur bacteria selected from in-dyked alluvial soil in hybrid maize fields. Note: Different letters indicate a significant difference (*p* < 0.05).

In general, the K-PNSB isolates solubilized better under ADC than MLC, except for isolates M-Sl-09 and M-So-11, whose P solubilization was equivalent between the two conditions. Under ADC, the isolates M-So-11 had the dissolved phosphate from Ca-P of 48.1 mg L^−1^ which was equivalent to those of the isolates M-So-25 and M-Sl-09 (46.8 and 46.6 mg L^−1^). The isolates M-Wa-07 and M-Sl-10 dissolved Ca-P with dissolved phosphate of 44.3 and 40.6 mg L^−1^). Moreover, the isolates M-So-14 had dissolved phosphate of 38.2 mg L^−1^. Furthermore, the isolates M-So-09 dissolved Ca-P the least, with 9.97 mg L^−1^. The other isolates had dissolved phosphate from Ca-P ranging from 11.1 to 36.6 mg L^−1^ (Figure 2). On the other hand, the isolates M-So-11 had the greatest dissolved Ca-P under MLC, with 6.965 mg L^−1^. The isolates M-Sl-09 had dissolved phosphate from Ca-P of 6394 mg L^−1^. In the meantime, the isolates M-So-14, M-Wa-11, and M-Sl-19 dissolved Ca-P at 0.737, 0.848, and 0.698 mg L^−1^, respectively. The other K-PNSB isolates poorly dissolved Ca-P, with dissolved phosphate ranging from 0.214 to 0.398 mg L^−1^.

Ultimately, according to the N fixation and Ca-P solubilization under ADC, then MLC, three isolates with the greatest performance were selected to investigate their capacity to produce plant-growth-promoting substances (IAA, ALA, siderophores, and EPS) and their genomic identity. They were M-Sl-09, M-So-11, and M-So-14.

#### 3.2.3. Production of IAA, ALA, Siderophores, and EPS by K-PNSB Isolates

**Figure 3:**
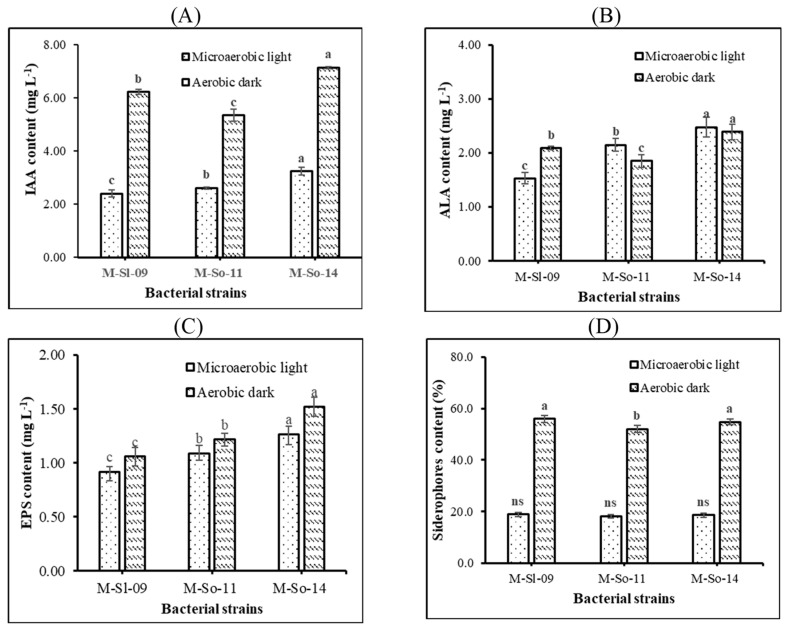
Production of plant-growth-promoting substances, including (**A**) indole-3-acetic acid, (**B**) 5-aminolevulinic acid, (**C**) exopolymeric substances, and (**D**) siderophores of the three isolates of potassium-dissolving purple nonsulfur bacteria selected from in-dyked alluvial soil in hybrid maize fields. Note: Different letters indicate a significant difference (*p* < 0.05). ns: not significant.

Between ADC and MLC, the IAA production and siderophores production by the three selected isolates showed remarkable differences, while the ALA production and EPS production did not exhibit such differences. In particular, the isolates M-So-14 produced IAA the most under both ADC and MLC, with 7.13 and 3.23 mg L^−1^. On the other hand, the ALA production of M-So-14 also peaked at 2.47 and 2.39 mg L^−1^, respectively. The isolates M-Sl-09 reached IAA and ALA contents of 6.22 and 2.09 mg L^−1^ under ADC, while the isolates M-So-11 produced IAA and ALA of 2.61 and 2.15 mg L^−1^ under MLC. However, the isolates M-Sl-09 produced IAA and ALA the least under MLC, with 2.40 and 1.53 mg L^−1^, respectively, while the isolates M-So-11 had the lowest IAA and ALA under ADC, with 5.34 and 1.85 mg L^−1^ (Figure 3A,B).

Additionally, the isolates M-So-14 produced EPS the most under both ADC and MLC, roughly 1.52 and 1.26 mg L^−1^. The isolates M-So-11 had EPS of 1.22 and 1.09 mg L^−1^ under ADC and MLC, which were greater than those of the isolate M-Sl-09, with 0.92 and 1.06 mg L^−1^ (Figure 3C). Moreover, the isolates M-Sl-09 and M-So-14 synthesized siderophores of 56.0 and 54.7% under ADC, which were greater than that of the isolate M-So-11, with 52.0%. The production of siderophores was equivalent among isolates under MLC, with an average siderophores content of 18.5% (Figure 3D).

### 3.3. Identification of K-PNSB Isolates from Hybrid Maize Farm

**Figure 4:**
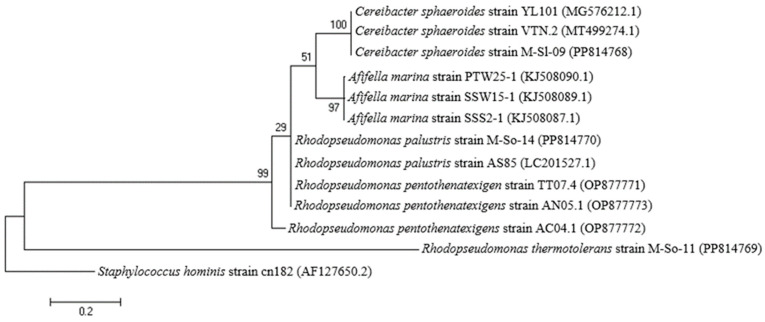
Neighbor-joining phylogenetic trees based on 16S rDNA sequences of two selected PNSB strains compared to the closely related strains in the GenBank database. The percentage levels of bootstrap analysis of 1000 replicates are indicated at each node. Bar, 0.2 substitutions per nucleotide position. Access numbers of GenBank sequences are implied in brackets.

**Figure 5:**
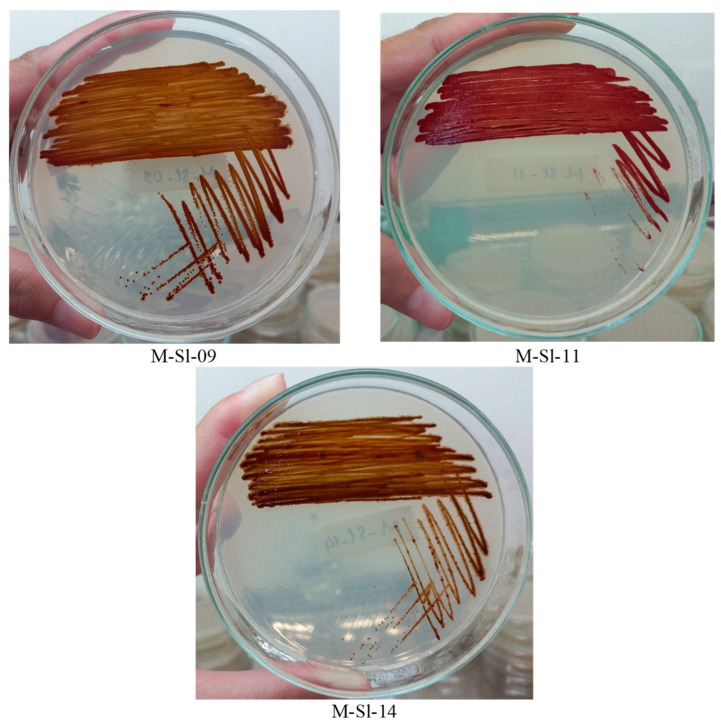
Colonies of the selected K-PNSB isolates *Cereibacter sphaeroides* M-Sl-09, *Rhodopseudomonas thermotolerans* M-So-11, and *Rhodospeudomonas palustris* M-So-14 on BIM.

The three selected K-PNSB isolates were identified as *Cereibacter sphaeroides* M-Sl-09, *Rhodopseudomonas thermotolerans* M-So-11, and *Rhodospeudomonas palustris* M-So-14, with a similarity of 99% (Figure 4). As can be seen, the three species belonged to distinct clusters, showing that the current study has successfully isolated three potent K-dissolvers. Moreover, their colonies are also shown in Figure 5 with brown and reddish-purple colors.

## 4. Discussion

In the current study, the pH_H2O_ was considered moderately acidic [36]. Electrical conductivity was from low to extremely low, which is suitable for vulnerable and moderately vulnerable plants [37]. However, the average exchangeable K concentration in the soil (0.267 ± 0.023 meq 100 g^−1^) was considered low according to Horneck et al. [38], i.e., the value was lower than 0.4 meq 100 g^−1^. This indicates the necessity of a K source for plants here. However, the application of chemical K fertilizer can lead to environmental pollution [39]. 

Fortunately, the current study presented three potent K-dissolvers. The three isolates M-Sl-09, M-So-11, and M-So-14 in this study can dissolve K under both ADC and MLC. This is consistent with the study by Khuong et al. [21], where K-PNSB isolates *Rhodopseudomonas pentothenatexigen* AC04.1 can dissolve K at 24.3 mg L^−1^ under ADC and 17.7 mg L^−1^ under MLC. Moreover, Liu et al. [40] claim that three isolates, *Bacillus megaterium* DD-2, *B. aryabhattai* DD-3, and *B. subtilis* DD-4, can dissolve K, with the greatest K_dis_ belonging to the isolate DD-4 (1.37 mg L^−1^). Sun et al. [41] have selected 18 KDB isolates from the roots of *Mikania micrantha,* which can dissolve K ranging from 0.07 to 1.75 mg L^−1^. The isolates *Bu. diffusa* HZ18, *Enterobacter hormaechei subsp. oharae* HZ9, and *Bu. diffusa* HZ23 can greatly dissolve K at 1.75, 1.25, and 1.17 mg L^−1^, respectively. Moreover, Fatharani and Rahayu [42] also state that seven isolates LJK 1, LJK 2, LJK 4, LBK 4, LBK 5, PSUK 4, and PSIK 6 obtained from paddy soils can dissolve K roughly 6.724–6.846 mg L^−1^ and are potent in applications as biofertilizers to alter chemical K fertilizer use. As per Zarjani et al. [43], KDB isolates *Arthrobacter* sp. JK2, *Bacillus megaterium* JK3, JK4, JK5, JK6, and JK7 were obtained from Mica-clay-mineral-rich soils and had K_dis_ of 910–1150 mg kg^−1^ under low pH conditions. Khanghahi et al. [44] have qualified 49 KDB isolates from 185 isolates from paddy soil, with three isolates *Pantoea agglomerans* KDB 37, *Rahnella aquatilis* KDB 39, and *Pseudomonas orientalis* KDB 44 which had the largest clear zones and greatest dissolution efficiency at 10.50, 9.00, and 10.83 mm and 1.64, 1.58, and 1.60 mm, respectively. Simultaneously, these three isolates have been quantified for their K-dissolving capacity with K_dis_ from Mica clay mineral in liquid Aleksandrov medium of 35.36, 76.04, and 56.58 μg mL^−1^ after 21 days of culture, and they have also been found to have potential for use as biofertilizers to improve soil exchangeable K to provide K for plants [44]. Khuong et al. [45] also say that the isolates *L. sphaeroides* EPS18, EPS37, and EPS54 can dissolve K under both ADC and MLC with 1.56–2.16 and 1.44–1.73 mg L^−1^, respectively. Ultimately, the results indicated the possibility of the current selected K-PNSB isolates being used as biofertilizers to replace the use of chemical fertilizers on the fields. This is a trend of sustainable agriculture [46].

Furthermore, in this study, the 3 K-PNSB isolates, M-Sl-09, M-So-11, and M-So-14, can fix nitrogen into available N (NH_4_^+^) and dissolve insoluble phosphate (Ca_3_(PO_4_)_2_) into soluble P for plants to take. This is in accordance with the study by Khuong et al. [21] in which the three K-PNSB isolates *R. pentothenatexigen* TT07.4, AN05.1, and AC04.1 can also fix nitrogen and dissolve Ca-P under ADC (7.93–11.2 mg L^−1^ and 20.2–25.1 mg L^−1^) and MLC (0.17–6.24 mg L^−1^ and 21.5–24.1 mg L^−1^). Liu et al. [40] reveal that apart from the K-dissolving ability, the isolates *B. megaterium* DD-2, *B. aryabhattai* DD-3, and *B. subtilis* DD-4 can also solubilize and mineralize insoluble phosphate. Khuong et al. [45] note that the three isolates *Luteovulum sphaeroides* EPS18, EPS37, and EPS54 can fix nitrogen (4.16–4.69 and 7.36–9.65 mg L^−1^) and dissolve Ca-P (11.8–13.7 and 15.6–18.9 mg L^−1^) under both ADC and MLC, respectively. This shows the applicability of the current K-PNSB isolates in replacing a portion of chemical N and P fertilizers used because they can make use of the N_2_ in the atmosphere and the immobilized P compounds in the soils. However, N and P are the most limiting nutrients to maize yield [47], while K may be easier to entirely replace by biofertilizers.

Apart from the nutrient-providing ability, the isolates M-Sl-09, M-So-11, and M-So-14 can also synthesize plant-growth-promoting substances such as IAA, EPS, ALA, and siderophores under ADC and MLC. According to Khuong et al. [21], the K-PNSB isolates *R. pentothenatexigens* TT07.4, AN05.1, and AC04.1 can produce IAA (12.9–32.6 and 13.6–17.8 mg L^−1^), EPS (0.14–0.76 and 0.21–0.86 mg L^−1^), ALA (0.63–3.01 and 1.19–6.39 mg L^−1^), and siderophores (28.4–30.3 and 6.15–10.3%) under MLC and ADC, respectively. This result had greater IAA and siderophores contents but lower ALA and EPS content than those in the current study (Figure 3). According to El-Egami et al. [48], *Bacillus circulans* can not only dissolve K (5.11 µg mL^−1^) but also produce IAA and siderophores (79.03 µg mL^−1^ and 11.42%, respectively). Furthermore, Khanghahi et al. [44] also stated that the isolate *Rahnella aquatilis* KDB 39 can produce IAA at 2.1 μg mL^−1^ and 6.6 μg mL^−1^ in media without or with L-tryptophan, respectively. As per Khuong et al. [49], *R. palustris* TLS06 and VNW64 produced IAA at 6.47–48.96 mg L^−1^ and 9.04–47.53 mg L^−1^ under MLC from day 1 to day 10 of incubation. Moreover, *R. palustris* TLS06 and VNW64 can also synthesize ALA at 3.43 and 3.35 mg L^−1^ under ADC and 2.96 and 3.41 mg L^−1^ under MLC after 10 days of incubation. Khuong et al. [50] claim that PNSB can also synthesize ALA under saline conditions (NaCl 1%) with ALA content of 0.43–4.61 mg L^−1^ under MLC and 0.29–3.94 mg L^−1^ under ADC. As reported by Khuong et al. [45], the three isolates *L. sphaeroides* EPS18, EPS37, and EPS54 produced IAA (2.14–2.56 and 2.96–3.16 mg L^−1^) and EPS (1.82–1.97 and 1.14–1.59 mg L^−1^) under ADC and MLC. Dat et al. [51] also investigated the production of IAA (10.3–21.0 mg IAA L^−1^), ALA (2.38–3.59 mg ALA L^−1^), siderophores (8.53–55.3%), and EPS (0.68–1.22 mg EPS L^−1^) by *Cereibacter* spp. ST16, ST26, ST27, and ST32 under ADC and MLC. Hence, the application of the *Cereibacter sphaeroides* M-Sl-09, *Rhodopseudomonas thermotolerans* M-So-11, and *Rhodospeudomonas palustris* M-So-14 isolates could improve the growth and yield of maize. Some KDB can secrete plant-growth-promoting substances [52]. For instance, the yield of crops, including rice and barley, was improved by the plant-growth-promoting substances produced by KDB [53,54]. Therefore, the K-PNSB in the current study is highly promising to be applicable and efficient in application to improve crop growth and yield. However, they should be further tested with different carriers under greenhouse conditions before a field trial. Choosing suitable carriers is of utmost importance because it depends on the real-world usage conditions [55]. Some potent carriers, such as rubber wood ash, decanter cake, rice husk ash, and spent coffee grounds, are suggested [56]. These should be further combined with the isolates in the current study.

## 5. Conclusions

Sixty-one K-PNSB isolates were obtained from in-dyked alluvial soil in hybrid maize fields in An Phu District, An Giang Province. Among them, 22 isolates had K_dis_ not lower than 40.0 mg L^−1^ under both MLC and ADC. From there, three K-PNSB isolates were selected for being able to fix nitrogen and solubilize P well under both MLC and ADC. These three isolates were identified as *Cereibacter sphaeroides* M-Sl-09, *Rhodopseudomonas thermotolerans* M-So-11, and *Rhodospeudomonas palustris* M-So-14. Moreover, the three isolates can also further synthesize IAA (5.34–7.13 and 2.40–3.23 mg L^−1^), ALA (1.85–2.47 and 1.53–2.39 mg L^−1^), siderophores (52.0–56.0 and 18.5%), and EPS (0.92–1.52 and 1.06–1.26%) under ADC and MLC, respectively. Ultimately, the current study has successfully isolated three promising K-dissolver and plant-growth promoters. Therefore, these three isolates should be further tested for their ability to replace chemical K fertilizer and to improve maize growth and yield in alluvial soils under greenhouse and later in field conditions.

## Figures and Tables

**Table 1:** Soil characteristics of the sampling locations at a depth of 0–20 cm to isolate potassium-dissolving purple nonsulfur bacteria.

Parameter	pH_H2O_	pH_KCl_	EC (µS cm^−1^)	CEC (meq 100 g^−1^)	Exchangeable K (meq 100 g^−1^)	Total N (%N)	NH_4_^+^ (mg kg^−1^)	Total P (%P_2_O_5_)	Soluble P (mg kg^−1^)	Ca-P (mg kg^−1^)
Highest	6.78	6.54	0.88	22.4	0.67	0.168	28.7	0.149	243.6	320.9
Median	5.70	4.93	0.35	19.6	0.24	0.112	9.62	0.089	98.0	177.0
Mean	5.73 ± 0.11	5.04 ± 0.15	0.39 ± 0.03	19.3 ± 0.36	0.27 ± 0.02	0.114 ± 0.005	9.23 ± 1.00	0.089 ± 0.004	108.9 ± 9.67	188.0 ± 8.57
Lowest	4.21	3.34	0.15	16.0	0.10	0.070	2.86	0.055	27.8	119.0

Note: EC: Electrical conductivity; CEC: cation exchange capacity.

**Table 2:** The dissolved K content by purple nonsulfur bacteria obtained from in-dyked alluvial soil in hybrid maize fields.

No.	Isolate	Dissolved K Content (mg L^−1^)	
		Aerobic Dark	Microaerobic Light
1	M-Sl-01	43.2 ^f–i^	49.0 ^ab^
2	M-Sl-02	48.5 ^ab^	45.6 ^cde^
3	M-Sl-06	38.7 ^m–p^	34.9 ^s–v^
4	M-Sl-07	41.7 ^h–l^	39.3 ^l–o^
5	M-Sl-08	37.7 ^op^	34.7 ^s–v^
6	M-Sl-09	48.1 ^ab^	49.6 ^a^
7	M-Sl-10	43.1 ^f–i^	42.5 ^g–k^
8	M-Sl-11	39.2 ^k–o^	37.2 ^o–s^
9	M-Sl-12	47.1 ^a–d^	41.1 ^i–m^
10	M-Sl-13	44.2 ^e–h^	42.8 ^f–j^
11	M-Sl-14	43.6 ^f–i^	44.1 ^d–g^
12	M-Sl-16	39.0 ^l–o^	34.9 ^s–v^
13	M-Sl-17	45.3 ^c–g^	43.3 ^e–i^
14	M-Sl-18	39.5 ^k–o^	34.8 ^s–v^
15	M-Sl-19	44.1 ^e–h^	41.8 ^g–l^
16	M-Sl-20	38.2 ^nop^	35.9 ^r–u^
17	M-Sl-21	42.5 ^g–j^	39.0 ^m–q^
18	M-Sl-23	41.9 ^h–k^	40.6 ^j–m^
19	M-So-02	39.3 ^k–o^	37.0 ^o–s^
20	M-So-06	39.6 ^k–o^	39.9 ^lmn^
21	M-So-07	43.6 ^f–i^	47.5 ^abc^
22	M-So-09	43.2 ^f–i^	43.8 ^e–h^
23	M-So-10	48.3 ^ab^	43.9 ^e–h^
24	M-So-11	48.8 ^a^	49.7 ^a^
25	M-So-14	48.8 ^a^	47.6 ^abc^
26	M-So-15	39.1 ^k–o^	40.2 ^k–n^
27	M-So-16	39.9 ^j–o^	37.7 ^n–r^
28	M-So-17	40.1 ^j–o^	36.5 ^p–t^
29	M-So-18	41.5 ^h–m^	39.9 ^l–n^
30	M-So-20	41.1 ^i–n^	39.2 ^l–p^
31	M-So-23	45.9 ^b–f^	43.3 ^e–i^
32	M-So-25	46.8 ^a–e^	43.9 ^e–h^
33	M-Wa-02	43.3 ^f–i^	34.0 ^tuv^
34	M-Wa-03	36.0 ^pq^	34.9 ^s–v^
35	M-Wa-05	37.6 ^opq^	32.5 ^vxy^
36	M-Wa-06	38.7 ^m–p^	31.5 ^xy^
37	M-Wa-07	47.8 ^abc^	42.8 ^f–j^
38	M-Wa-08	44.8 ^d–g^	41.3 ^h–m^
39	M-Wa-10	39.8 ^j–o^	30.7 ^y^
40	M-Wa-11	44.8 ^d–g^	48.4 ^ab^
41	M-Wa-15	35.0 ^q^	35.5 ^r–u^
42	M-Wa-17	39.9 ^j–o^	36.4 ^q–t^
43	M-Wa-19	37.2 ^opq^	34.1 ^tuv^
44	M-Wa-20	44.4 ^e–h^	46.5 ^bcd^
45	M-Wa-22	45.9 ^b–f^	45.3 ^c–f^
46	M-Wa-23	39.2 ^k–o^	39.0 ^m–q^
47	M-Wa-25	37.6 ^opq^	33.5 ^uvx^
48	M-Wa-26	39.4 ^k–o^	36.9 ^o–s^
Level of significance		*	*
CV (%)		3.50	3.45

Note: In the same column, numbers followed by different letters are different at 5% significance (*).

## Data Availability

The original contributions presented in the study are included in the article/Supplementary Material; further inquiries can be directed to the corresponding author.

## Supplementary Materials

The following supporting information can be downloaded at: https://www.mdpi.com/article/10.3390/life14111461/s1, Figure S1: Sampling locations to isolate purple nonsulfur bacteria in An Phu District, An Giang Province; Figure S2: Morphology of 61 potassium-dissolving purple nonsulfur bacteria on Petri dishes; Table S1: Dissolved potassium content by the potassium-dissolving purple nonsulfur bacteria selected from in-dyked alluvial soil in hybrid maize fields in An Phu District, An Giang Province under pH 5.5 conditions.
