# Supplementary material for: Characterization of Novel Species of Potassium-Dissolving Purple Nonsulfur Bacteria Isolated from In-Dyked Alluvial Upland Soil for Maize Cultivation
Source: Life (Basel). 2024 Nov 12;14(11):1461. doi: 10.3390/life14111461 (PMC11595535; doi:10.3390/life14111461)
Supplement: Supplementary file 1 [file life-14-01461-s001.zip › life-3060962-supplementary.pdf]

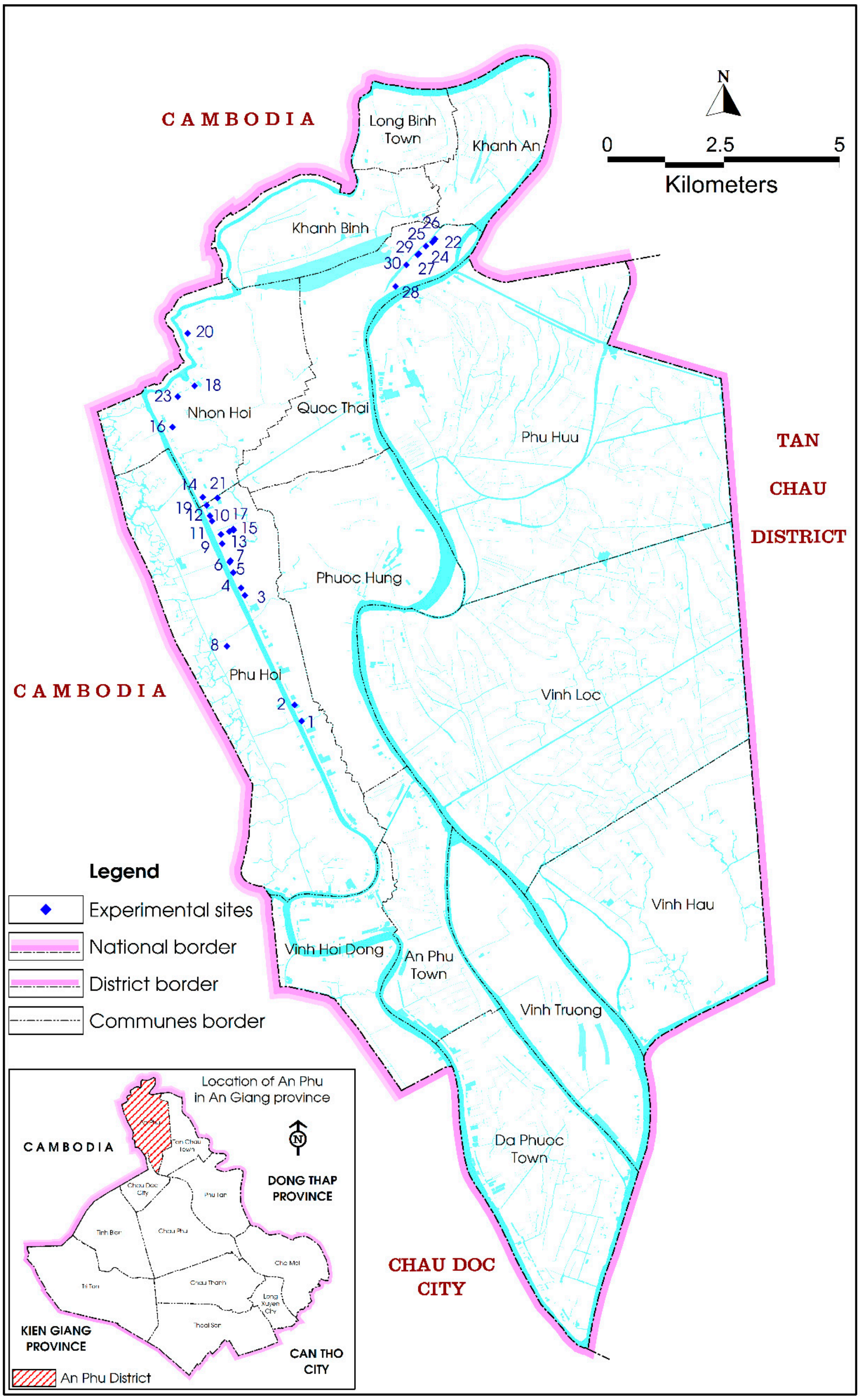

Figure S1. Sampling locations to isolate purple nonsulfur bacteria in An Phu District, An Giang Province

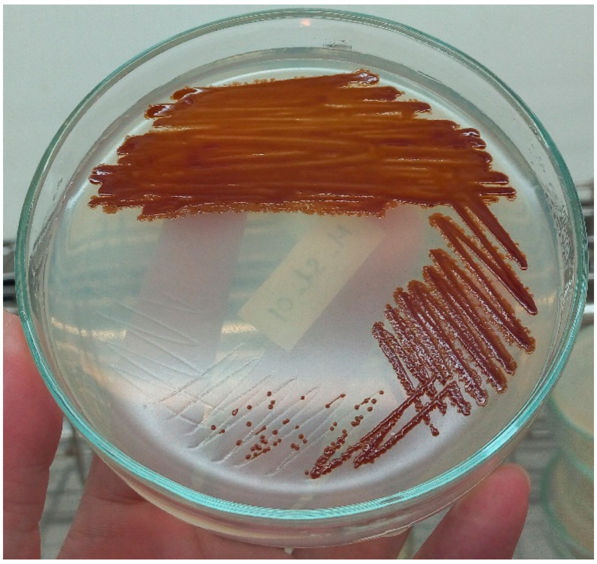

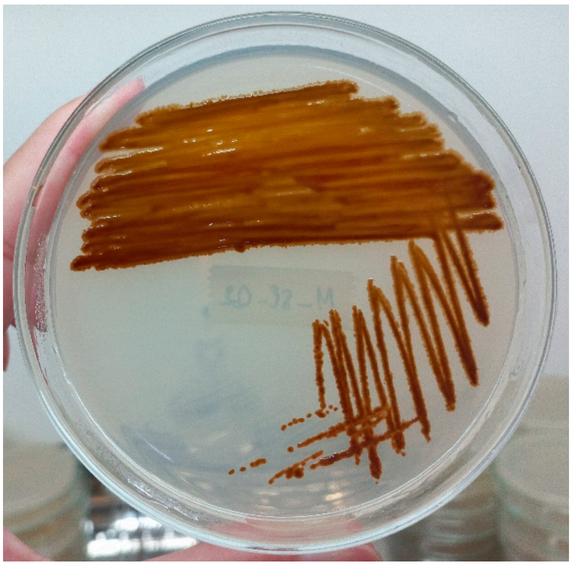

M-Sl-01

M-Sl-02

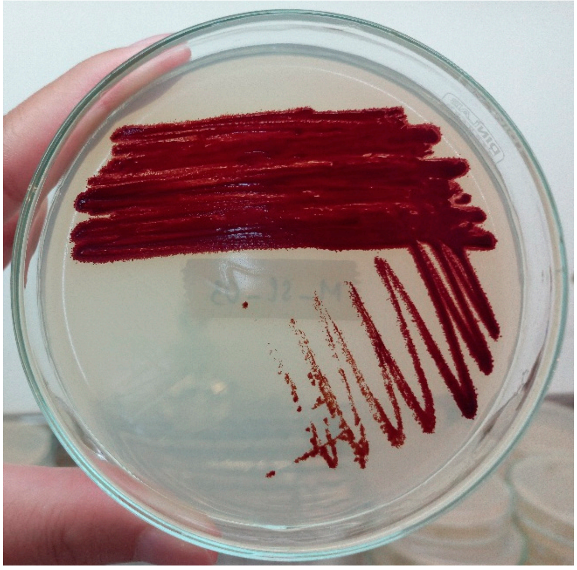

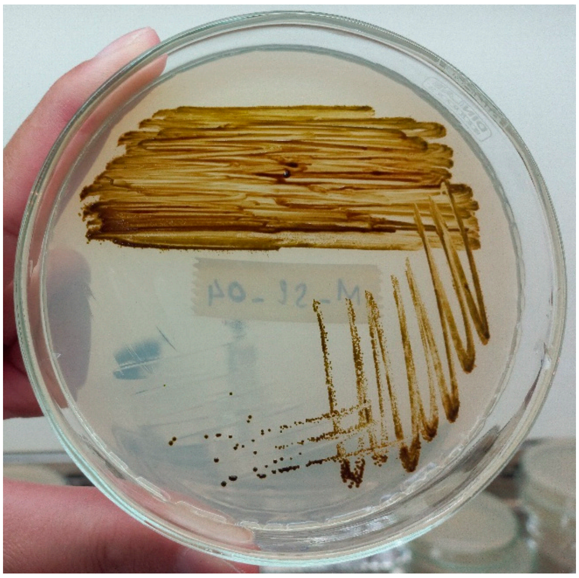

M-Sl-03

M-Sl-04

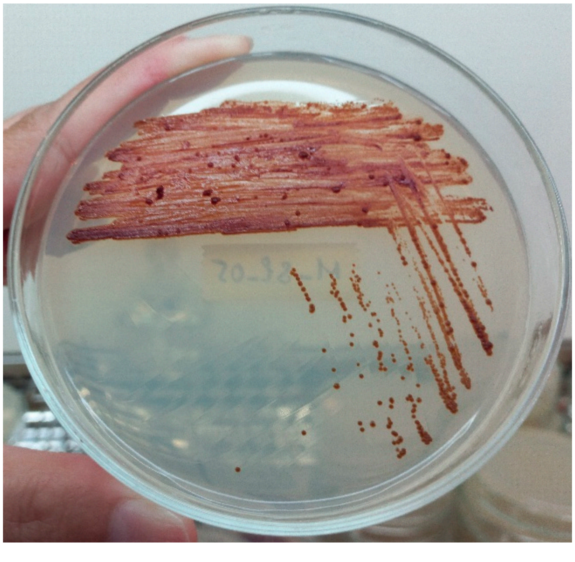

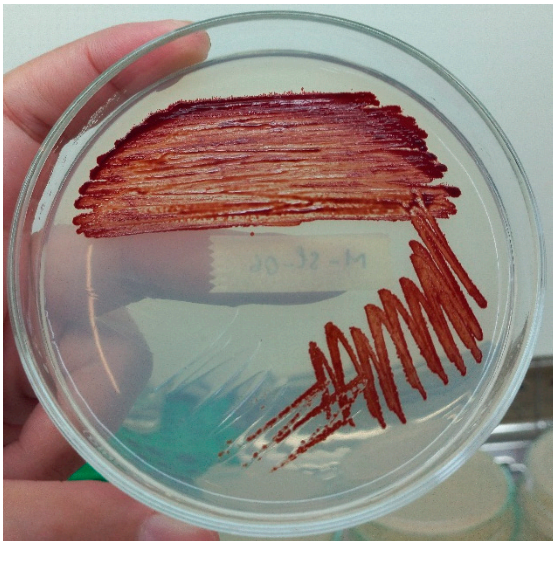

M-Sl-05

M-Sl-06

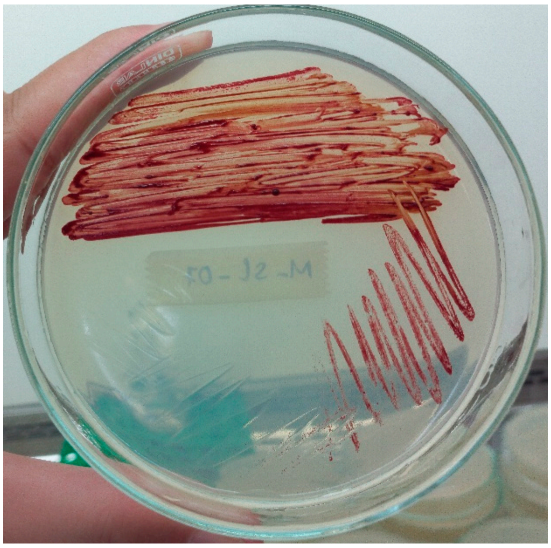

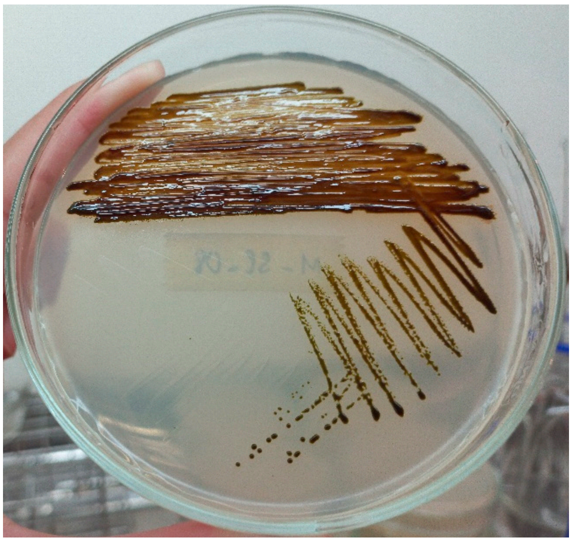

M-Sl-07

M-Sl-08

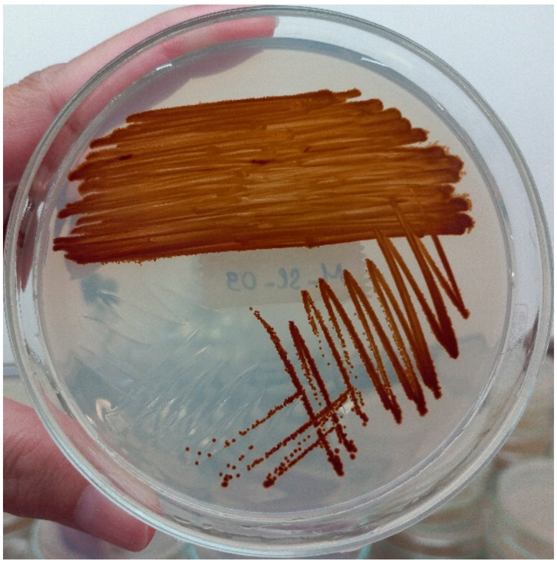

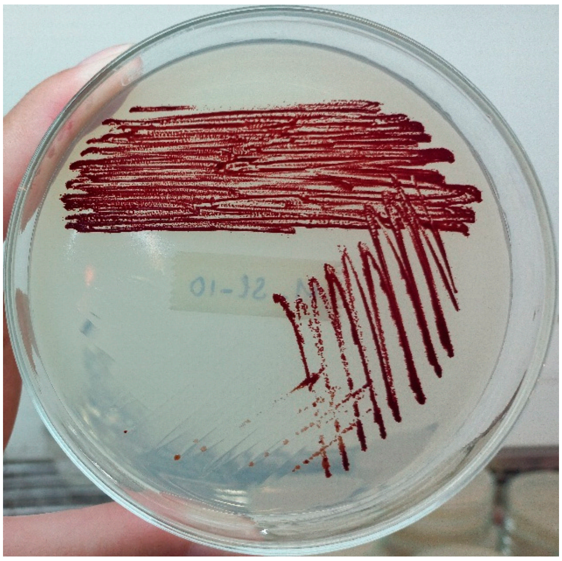

M-Sl-09

M-Sl-10

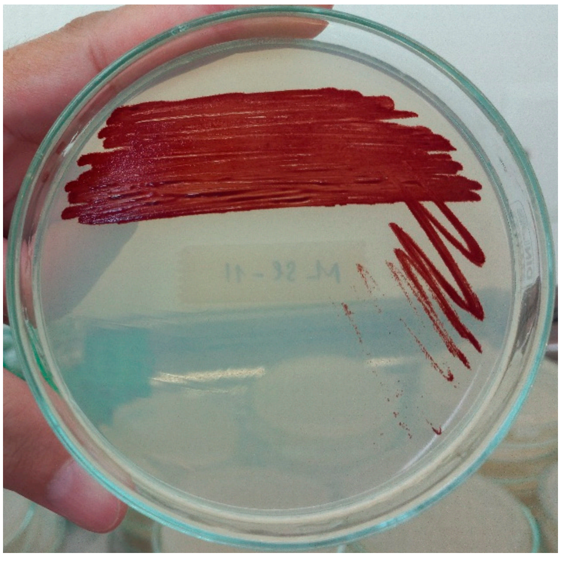

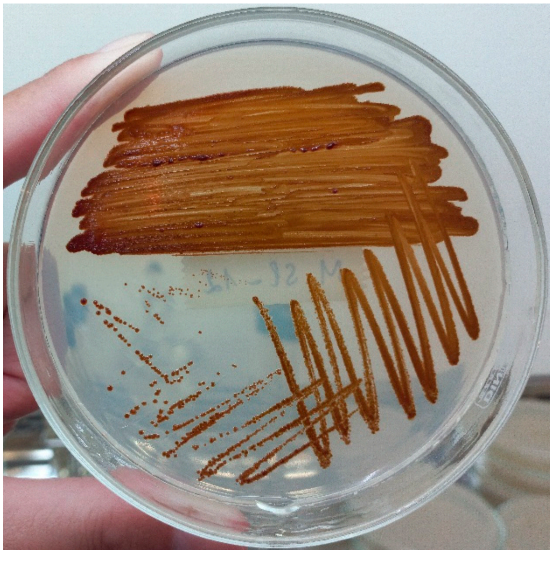

M-Sl-11

M-Sl-12

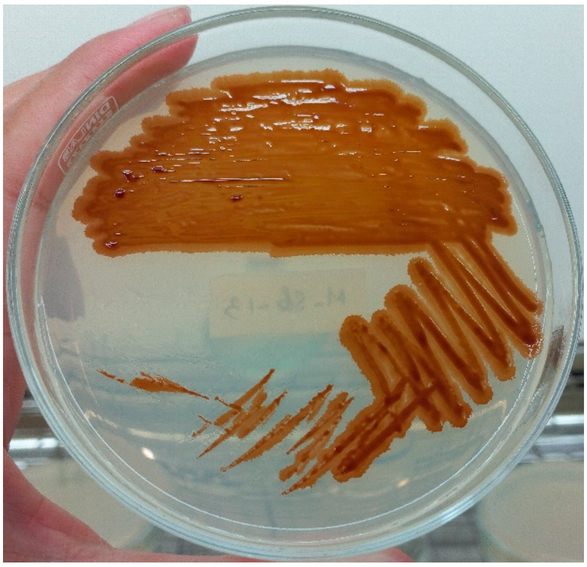

M-Sl-13

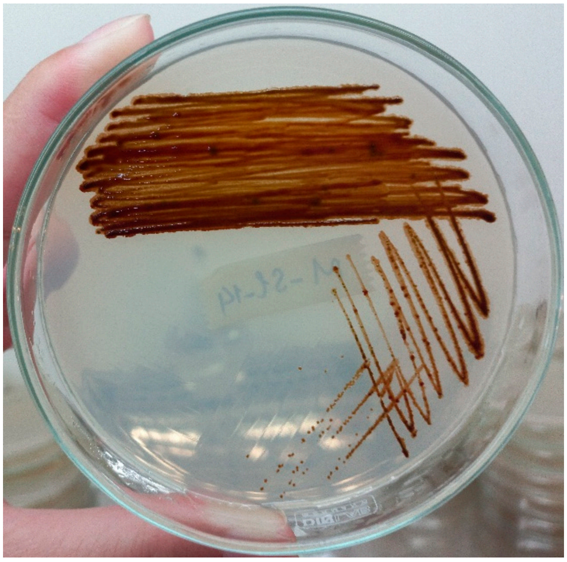

M-Sl-14

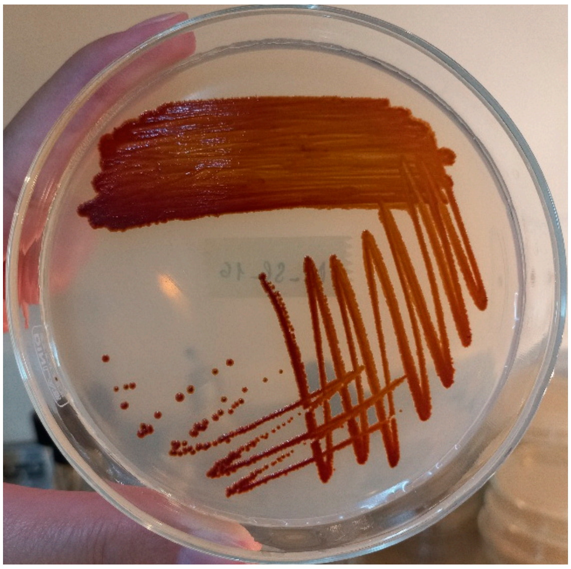

M-Sl-16

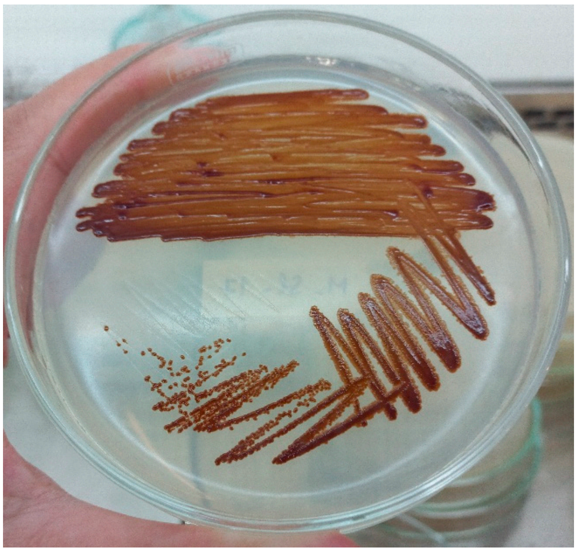

M-Sl-17

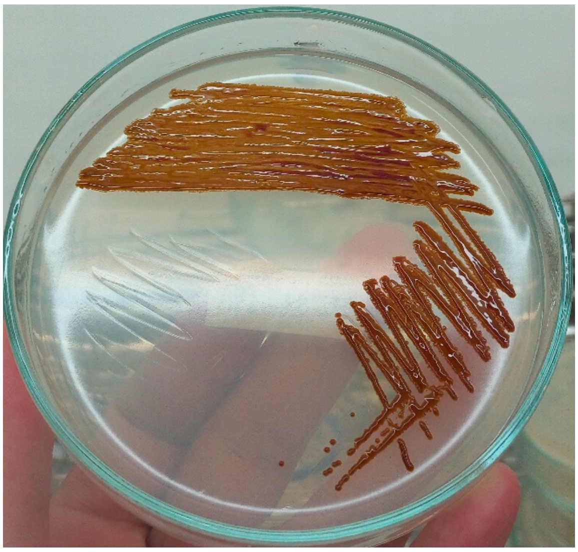

M-Sl-18

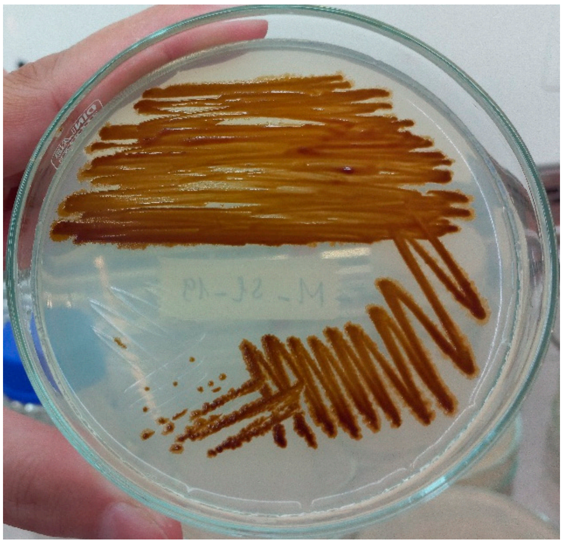

M-Sl-19

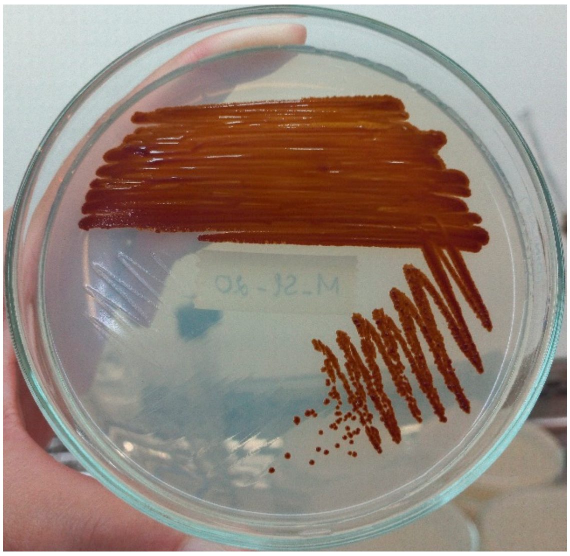

M-Sl-20

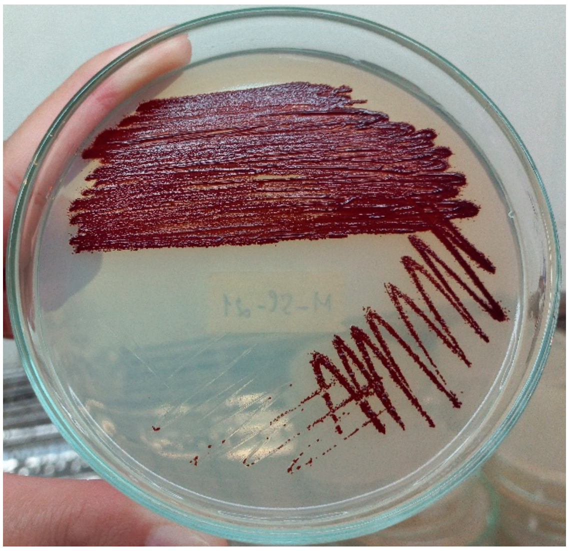

M-Sl-21

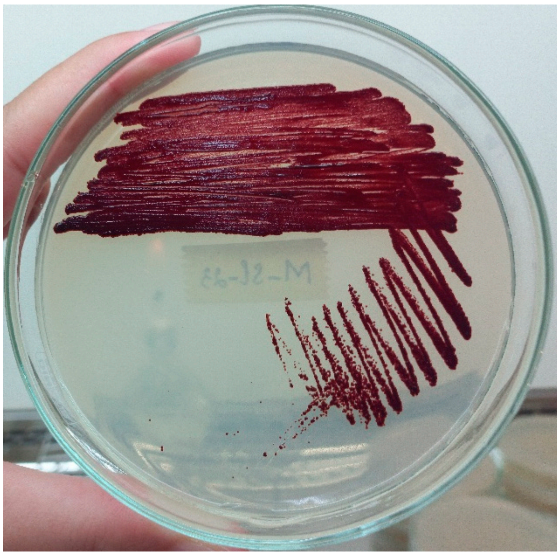

M-Sl-23

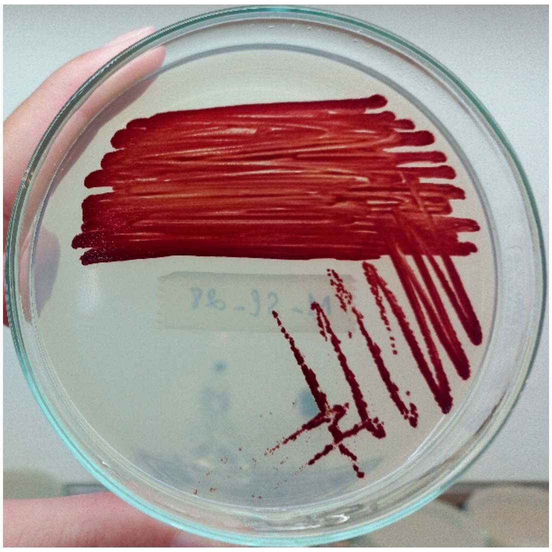

M-Sl-28

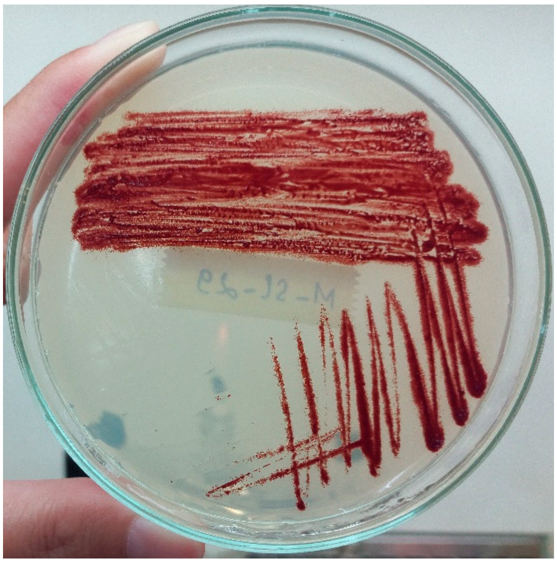

M-Sl-29

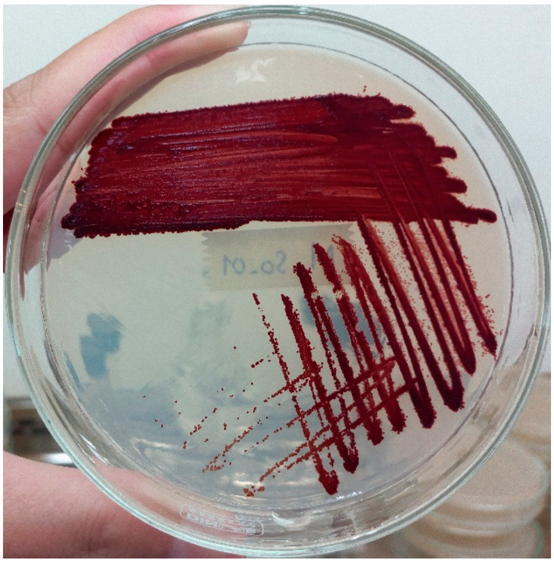

M-So-01

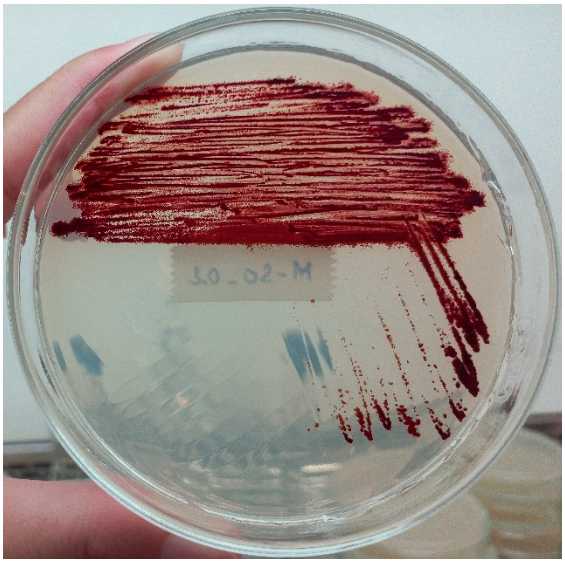

M-So-02

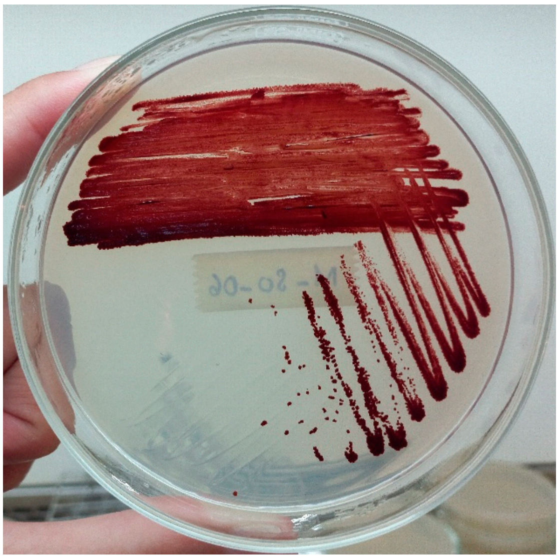

M-So-06

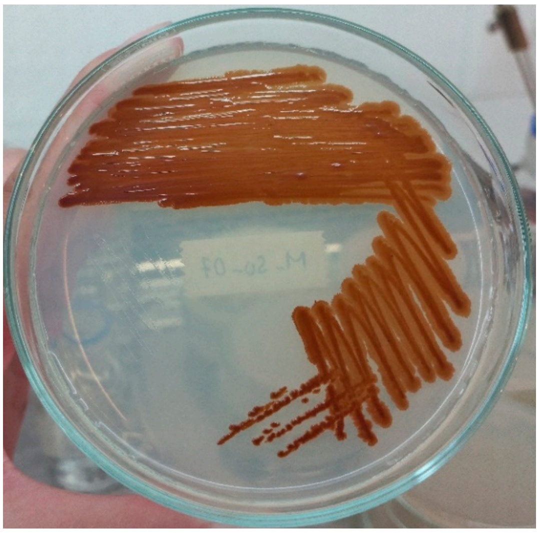

M-So-07

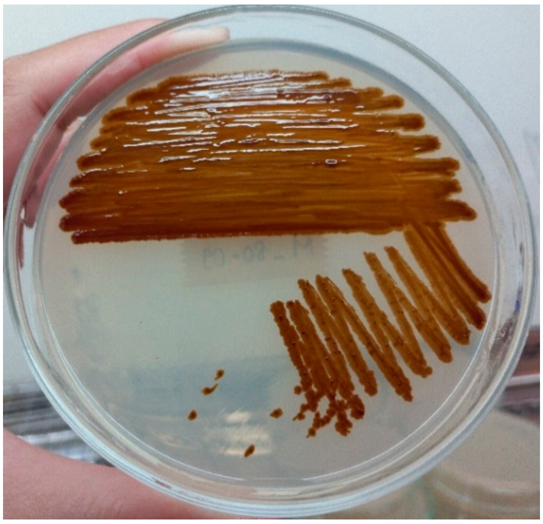

M-So-09

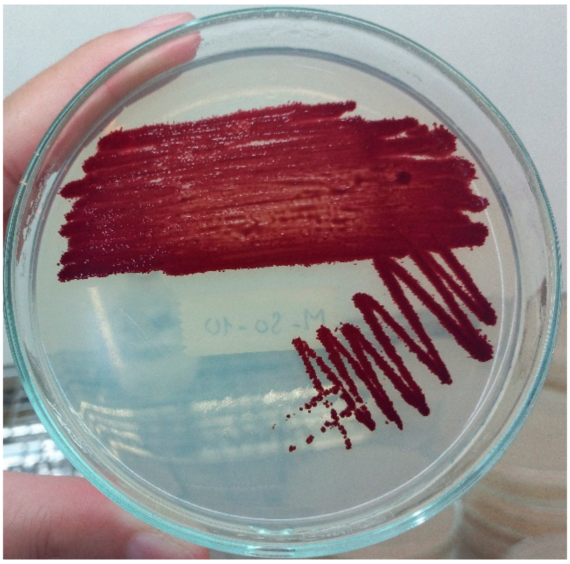

M-So-10

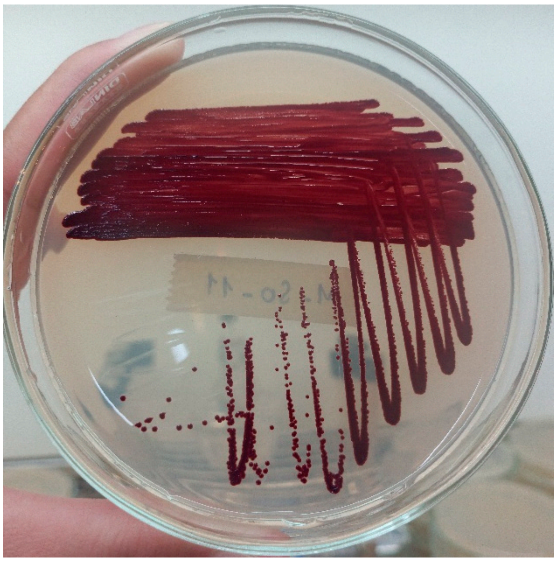

M-So-11

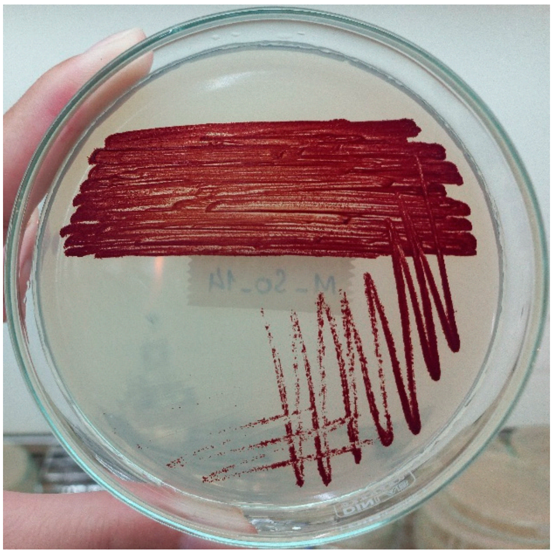

M-So-14

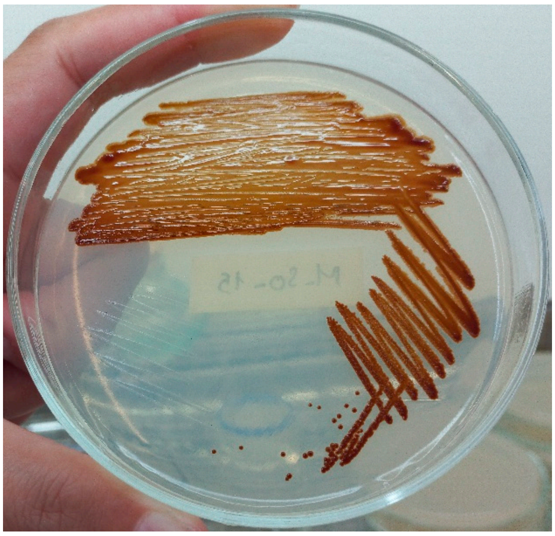

M-So-15

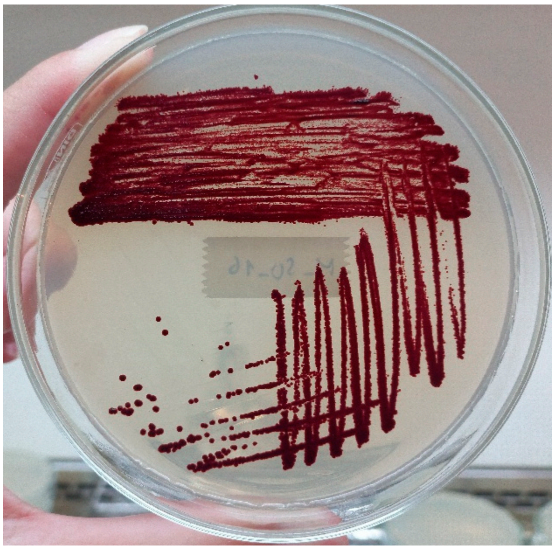

M-So-16

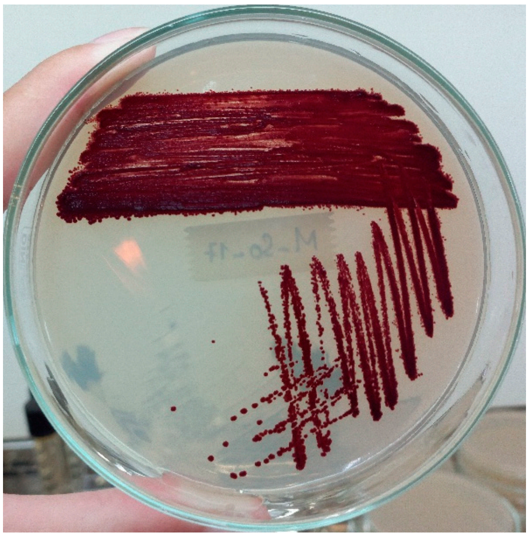

M-So-17

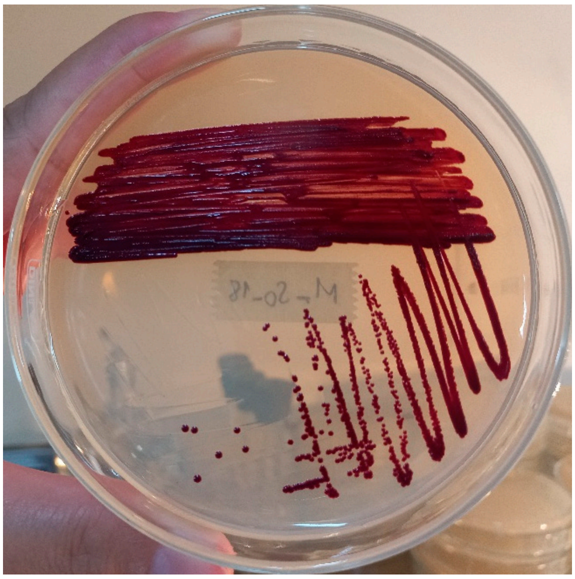

M-So-18

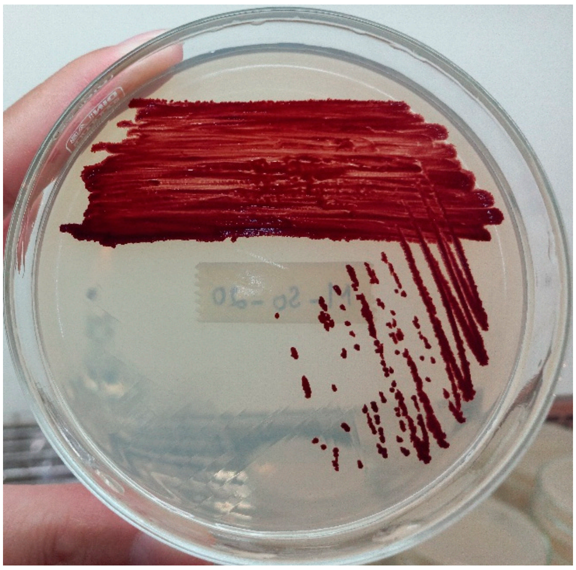

M-So-20

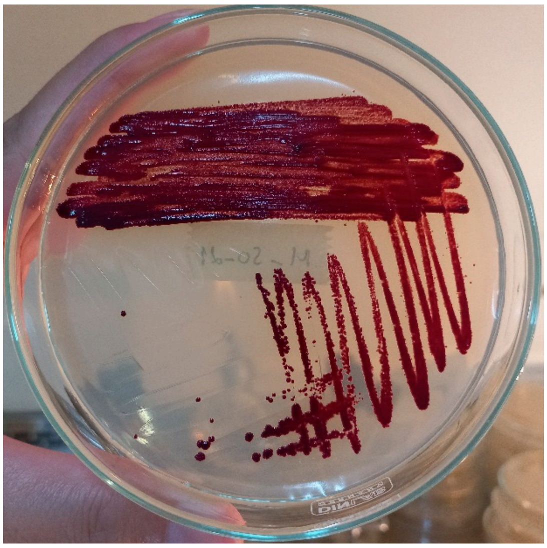

M-So-21

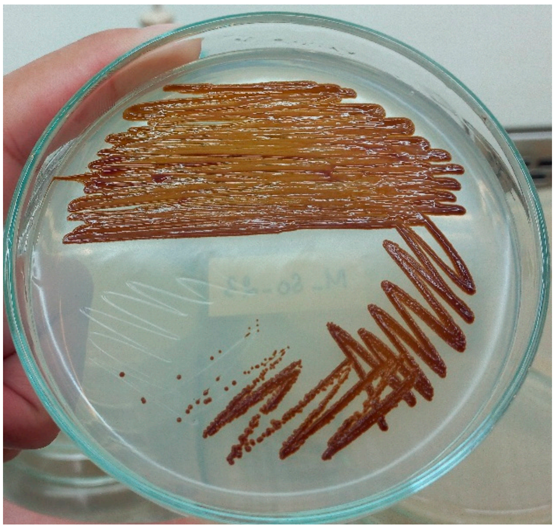

M-So-23

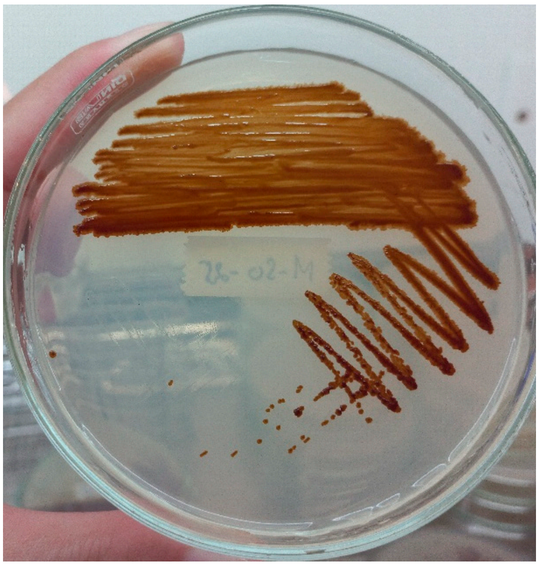

M-So-25

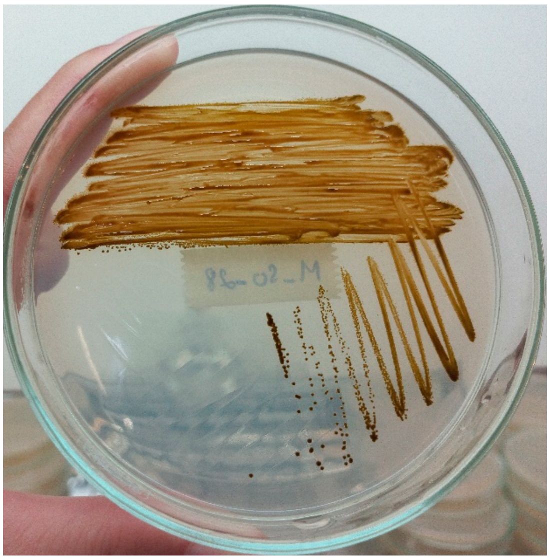

M-So-28

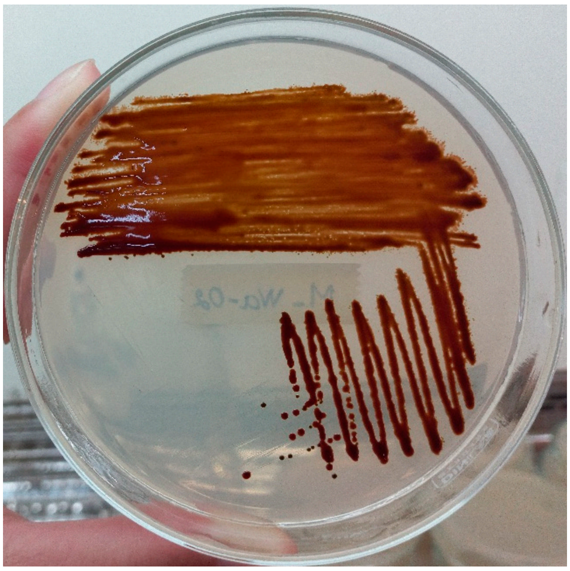

M-Wa-02

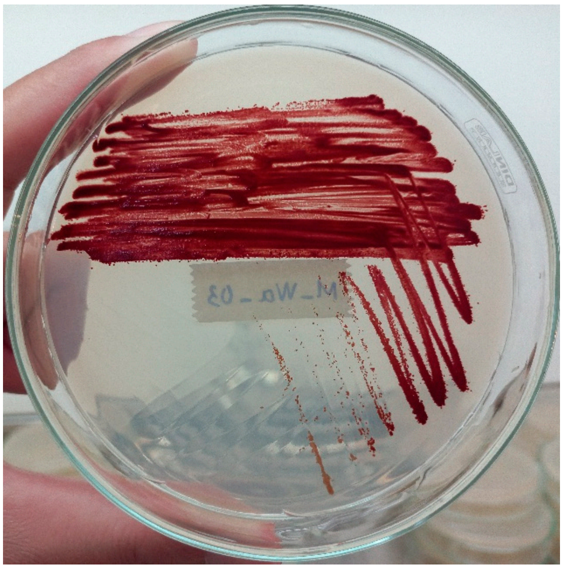

M-Wa-03

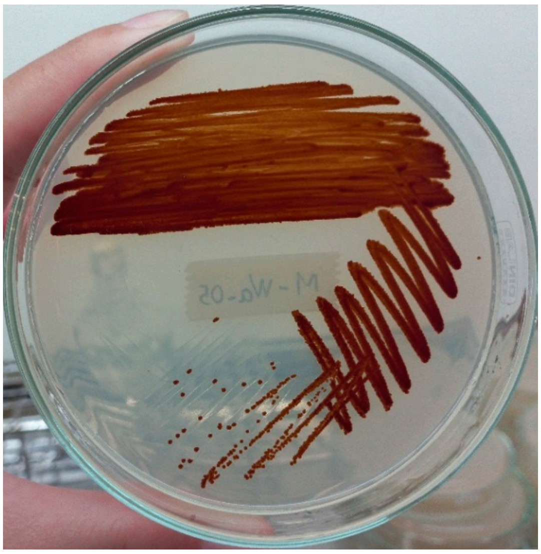

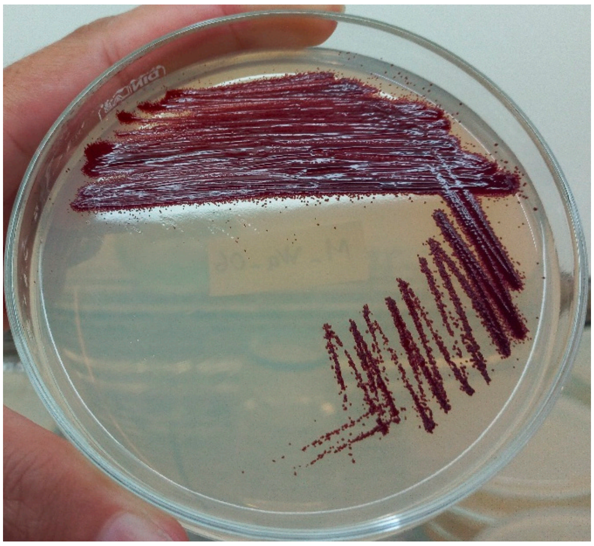

M-Wa-05

M-Wa-06

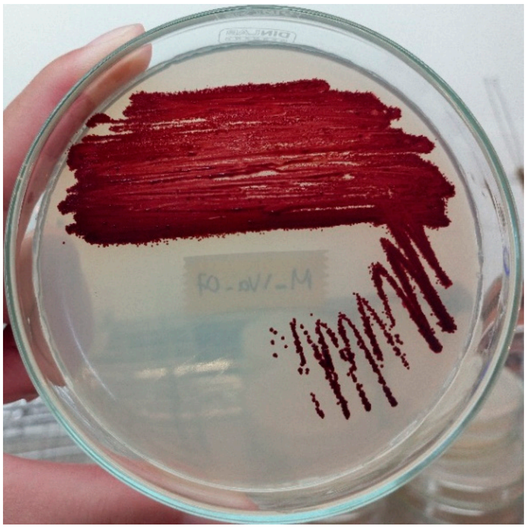

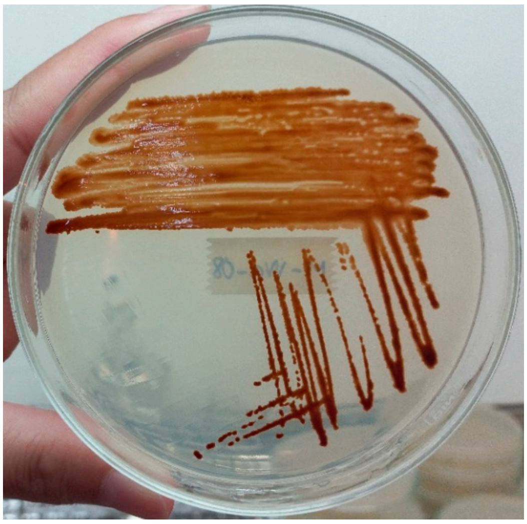

M-Wa-07

M-Wa-08

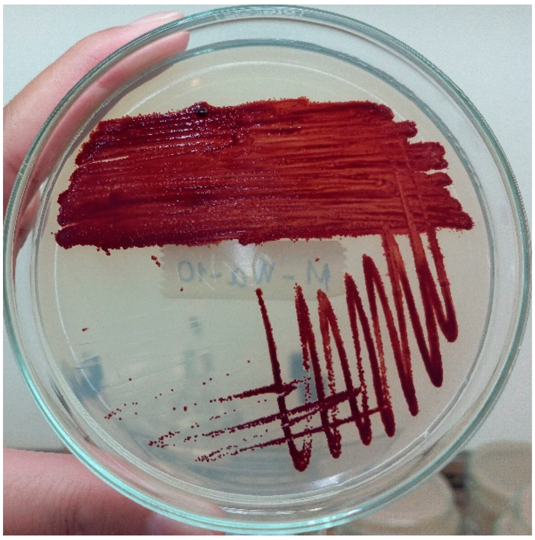

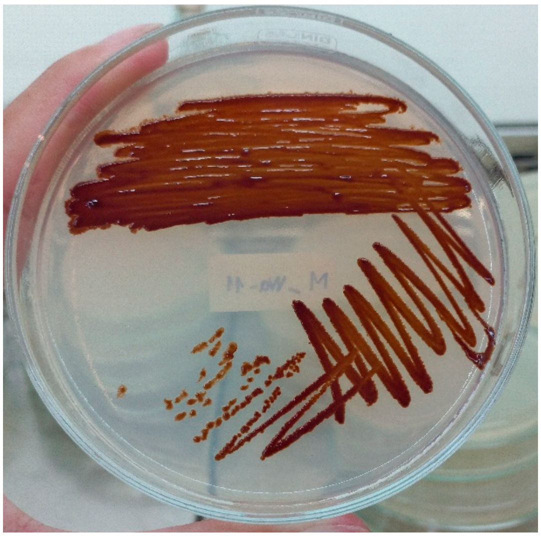

M-Wa-10

M-Wa-11

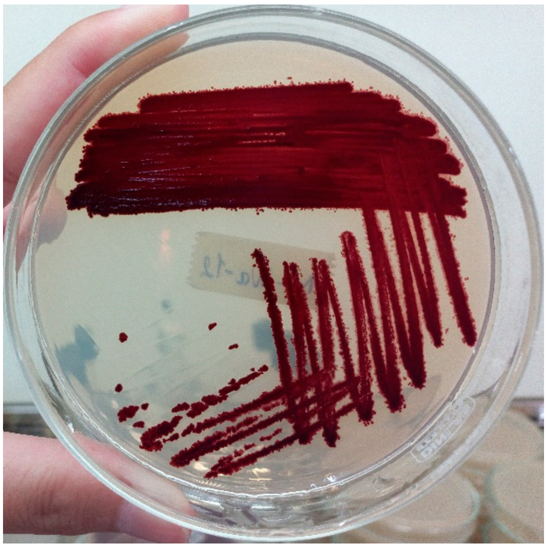

M-Wa-12

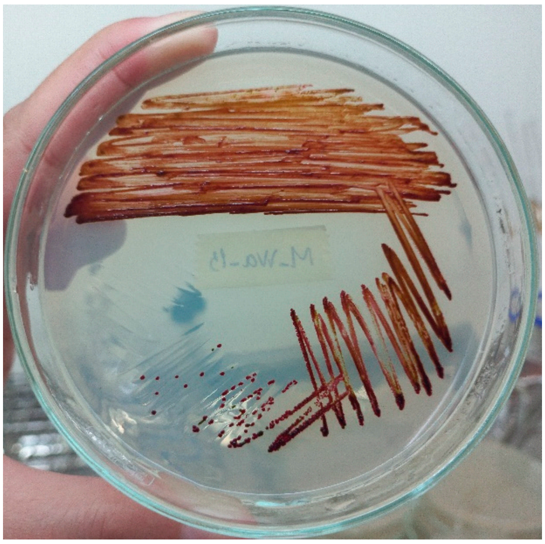

M-Wa-13

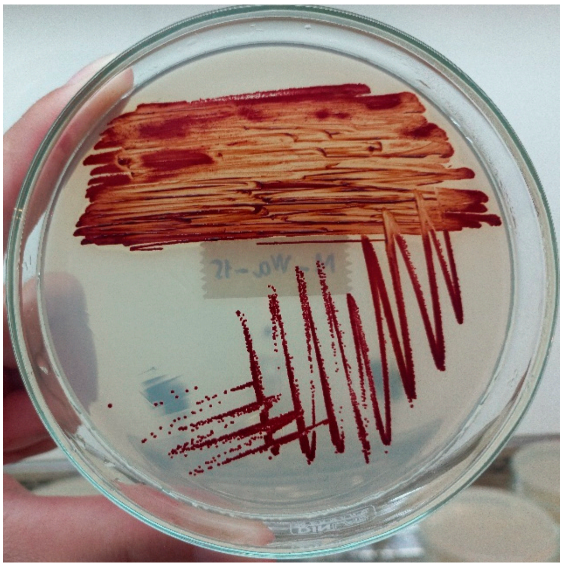

M-Wa-15

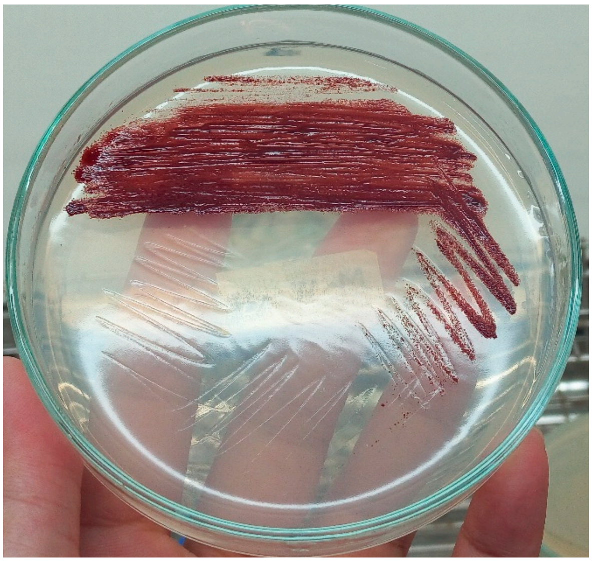

M-Wa-17

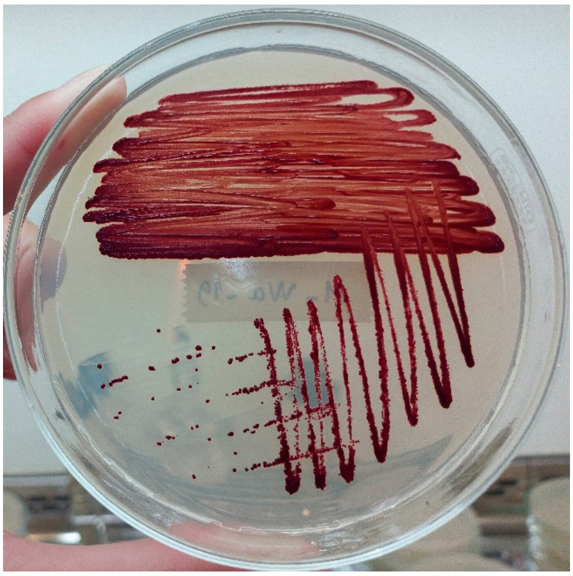

M-Wa-19

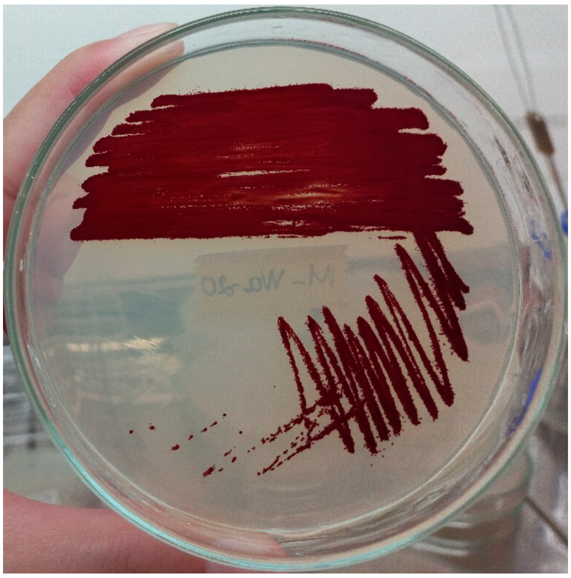

M-Wa-20

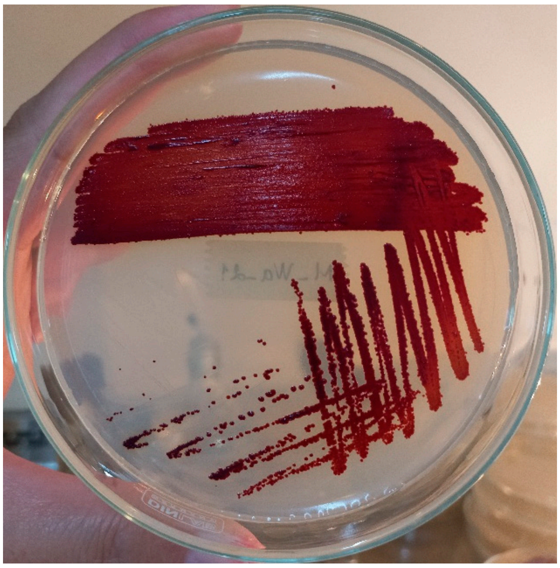

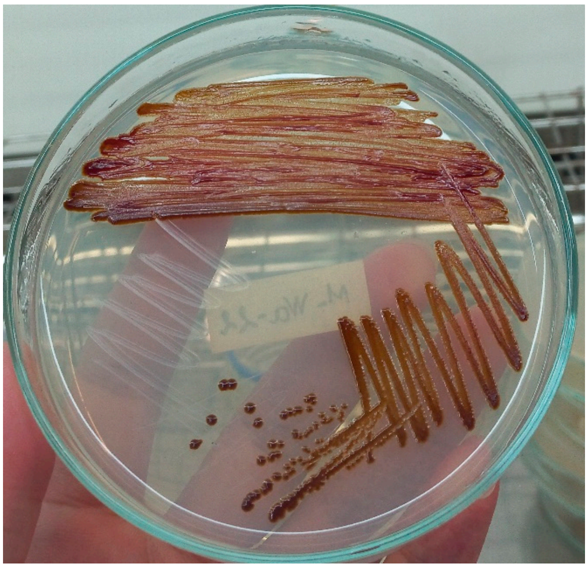

M-Wa-21

M-Wa-22

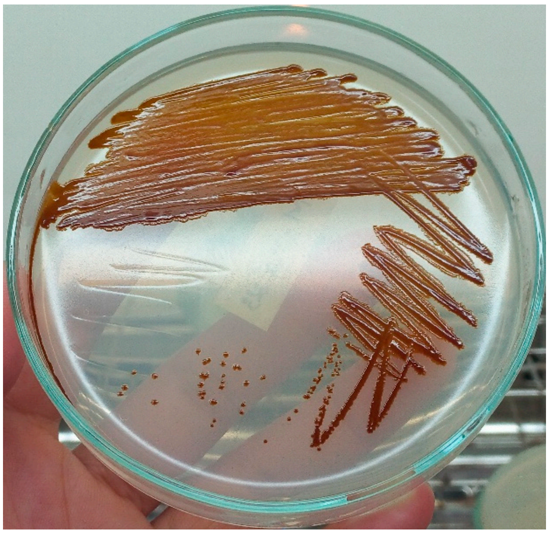

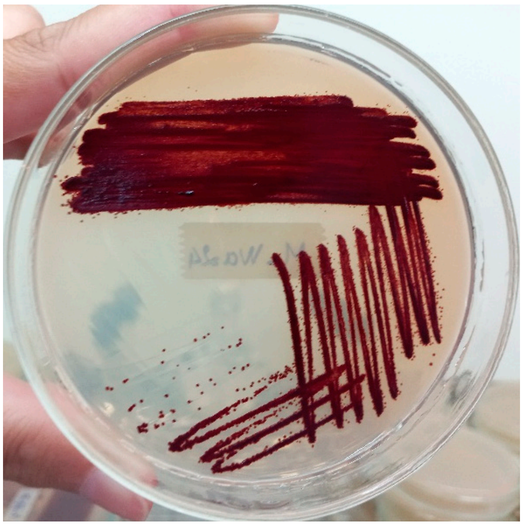

M-Wa-23

M-Wa-24

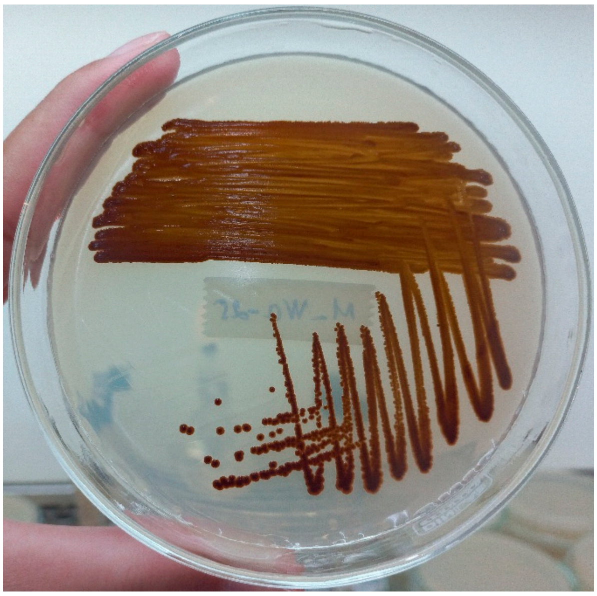

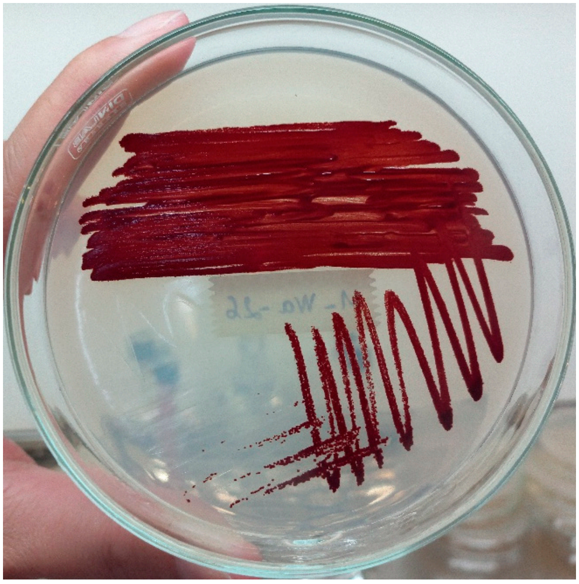

M-Wa-25

M-Wa-26

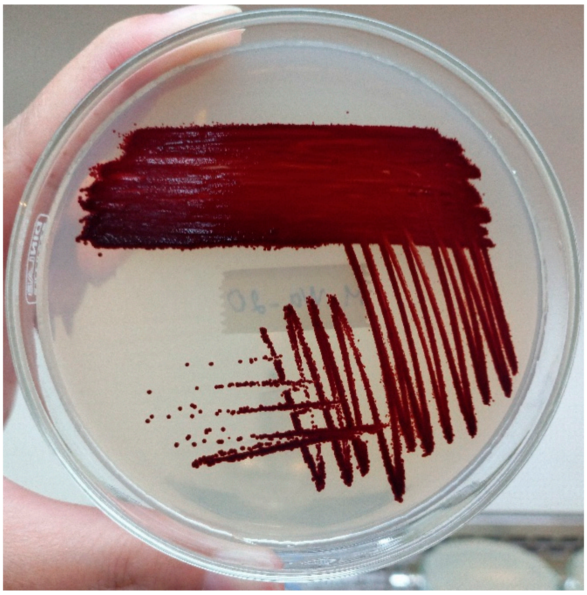

M-Wa-30

Figure S2. Morphology of 61 potassium-dissolving purple nonsulfur bacteria on Petri dishes**.**

Table S1. Dissolved potassium content by the potassium-dissolving purple nonsulfur bacteria selected from in-dyked alluvial soil in hybrid maize fields in An Phu District, An Giang Province under pH 5.5 conditions.

No.	Isolate	Dissolved potassium content (mg L^{-1})		
		Aerobic dark	Microaerobic light	Total
1	M-So-11	49.7	48.8	98.6
2	M-Sl-09	49.6	48.1	97.7
3	M-So-14	47.6	48.8	96.4
4	M-Sl-02	45.6	48.5	94.1
5	M-Wa-11	48.4	44.8	93.2
6	M-So-10	43.9	48.3	92.2
7	M-Sl-01	49.0	43.2	92.2
8	M-Wa-22	45.3	45.9	91.2
9	M-So-07	47.5	43.6	91.1
10	M-Wa-20	46.5	44.4	90.9
11	M-So-25	43.9	46.8	90.8
12	M-Wa-07	42.8	47.8	90.6
13	M-So-23	43.3	45.9	89.2
14	M-Sl-17	43.3	45.3	88.6
15	M-Sl-12	41.1	47.1	88.3
16	M-Sl-14	44.1	43.6	87.7
17	M-Sl-13	42.8	44.2	87.1
18	M-So-09	43.8	43.2	86.9
19	M-Wa-08	41.3	44.8	86.1
20	M-Sl-19	41.8	44.1	85.8
21	M-Sl-10	42.5	43.1	85.7
22	M-Sl-23	40.6	41.9	82.5
23	M-Sl-21	39.0	42.5	81.5
24	M-So-18	39.9	41.5	81.4
25	M-Sl-07	39.3	41.7	81.0
26	M-So-20	39.2	41.1	80.3
27	M-So-06	39.9	39.6	79.5
28	M-So-15	40.2	39.1	79.3
29	M-Wa-23	39.0	39.2	78.2
30	M-So-16	37.7	39.9	77.6
31	M-Wa-02	34.0	43.3	77.3
32	M-So-17	36.5	40.1	76.6
33	M-Wa-17	36.4	39.9	76.3
34	M-Wa-26	36.9	39.4	76.3
35	M-Sl-11	37.2	39.2	76.3
36	M-So-02	37.0	39.3	76.3
37	M-Sl-18	34.8	39.5	74.2
38	M-Sl-20	35.9	38.2	74.1
39	M-Sl-16	34.9	39.0	73.9

40	M-Sl-06	34.9	38.7	73.5
41	M-Sl-08	34.7	37.7	72.4
42	M-Wa-19	34.1	37.2	71.4
43	M-Wa-25	33.5	37.6	71.1
44	M-Wa-03	34.9	36.0	70.9
45	M-Wa-10	30.7	39.8	70.5
46	M-Wa-15	35.5	35.0	70.5
47	M-Wa-06	31.5	38.7	70.2
48	M-Wa-05	32.5	37.6	70.1
49	M-So-21	32.3	33.5	65.7
50	M-So-01	29.2	36.7	65.9
51	M-Wa-30	26.1	38.5	64.6
52	M-Sl-29	35.2	30.2	65.6
53	M-Sl-28	30.1	35.8	65.9
54	M-Wa-24	36.8	31.9	68.7
55	M-Wa-21	31.5	32.9	64.3
56	M-So-28	31.3	36.5	67.8
57	M-Sl-04	34.3	33.5	67.7
58	M-Wa-13	30.9	32.3	63.2
59	M-Wa-12	35.5	30.5	66.0
60	M-Sl-05	32.5	26.9	59.4
61	M-Sl-03	27.0	29.2	56.2

Note: M-maize, Wa-water, Sl-slurry, and So-soil
